# OTUD7B Activates the Caspase-8-RIPK1-NEMO Complex-Regulated NF-κB Pathway to Promote Triple-Negative Breast Cancer Metastasis

**DOI:** 10.32604/or.2026.081093

**Published:** 2026-09-14

**Authors:** Fiona Tsui-Fen Cheng, Kung-Ju Chen, Jing-Quan Zheng, Hui-Wen Chiu, Hui-Yu Lin, Yuan-Feng Lin

**Affiliations:** 1 Breast Cancer Center, Shin Kong Wu Ho-Su Memorial Hospital, Taipei, Taiwan; 2 School of Medicine, Fu-Jen Catholic University, New Taipei City, Taiwan; 3 Graduate Institute of Clinical Medicine, College of Medicine, Taipei Medical University, Taipei, Taiwan; 4 Division of Pulmonary Medicine, Department of Internal Medicine, Shuang Ho Hospital, Taipei Medical University, New Taipei City, Taiwan; 5 Division of Pulmonary Medicine, Department of Internal Medicine, School of Medicine, College of Medicine, Taipei Medical University, Taipei, Taiwan; 6 Department of Medical Research, Shuang Ho Hospital, Taipei Medical University, New Taipei City, Taiwan; 7 TMU Research Center of Urology and Kidney, Taipei Medical University, Taipei, Taiwan; 8 Division of Breast Surgery and General Surgery, Department of Surgery, Cardinal Tien Hospital, Fu-Jen Catholic University, New Taipei City, Taiwan; 9 Cell Physiology and Molecular Image Research Center, Wan Fang Hospital, Taipei Medical University, Taipei, Taiwan

**Keywords:** Triple-negative breast cancer (TNBC), metastasis, OTUD7B, Caspase-8, NEMO, NF-κB

## Abstract

**Background:** Metastatic dissemination of triple-negative breast cancer (TNBC) to distant organs, such as the lungs and brain, poses a significant threat to patient survival. Nevertheless, the molecular basis driving TNBC metastasis remains largely elusive. In the present study, we elucidated the role and underlying mechanism of OTU deubiquitinase 7B (OTUD7B) in promoting TNBC metastasis. **Methods:** The Cancer Genome Atlas (TCGA)/K-M Plotter databases were used for determining the prognostic significance of OTUD7B in TNBC patients. Cell migration and lung colony-forming assays were performed to evaluate the metastatic potential of TNBC cells. A cycloheximide-chase assay was employed to examine the effect of OTUD7B on Nuclear factor kappa-light-chain-enhancer of activated B cells (NF-κB) Essential Modulator (NEMO) protein degradation in TNBC cells. Flow-cytometric analyses were performed to examine OTUD7B effects on TNBC necroptosis. **Results:** OTUD7B is significantly (*p* < 0.001) upregulated in TNBC and correlates with poor distant metastasis-free survival (log-rank *p* < 0.001, *n* = 424). Its knockdown suppresses, whereas overexpression enhances, the metastatic potential of TNBC cells *in vitro* and *in vivo*, dependent on its deubiquitinating activity. Mechanistically, OTUD7B regulates the ubiquitination of Caspase-8 and NEMO, thereby modulating NF-κB signaling through the Caspase-8–RIPK1–NEMO axis. OTUD7B depletion increases Caspase-8 activity by approximately three-fold, promotes NEMO degradation, suppresses NF-κB activation, and induces necroptosis. Conversely, OTUD7B overexpression exerts opposite effects. Pharmacological inhibition of NEMO or NF-κB attenuates OTUD7B-driven cell migration by 40–90%. **Conclusions:** The deubiquitinating activity of OTUD7B promotes TNBC metastasis by stabilizing the Caspase-8–RIPK1–NEMO axis, thereby activating the NF-κB signaling pathway. These results further suggest that targeting OTUD7B activity may be a promising therapeutic strategy for metastatic TNBC.

## 1 Introduction

Triple-negative breast cancer (TNBC) is an immunohistochemically defined subtype of breast cancer (BC) characterized by the absence of estrogen receptor (ER), progesterone receptor (PR), and human epidermal growth factor receptor 2 (HER2) expression and is associated with a poor prognosis, likely because of its highly metastatic nature [1]. On the basis of gene expression profiling, BC can be further categorized into five intrinsic molecular subtypes: luminal A, luminal B, HER2-enriched, normal-like, and basal-like [2,3]. Approximately 75% of basal-like breast cancers are TNBCs. A previous report on the metastatic behavior of BC subtypes demonstrated that bone metastasis was common across all non-basal-like subtypes, whereas the basal-like subtype had higher rates of brain, lung, and distant nodal metastases [4]. Accordingly, elucidating the molecular mechanisms that govern TNBC metastasis is essential for enabling more precise and effective management of this disease.

OTUD7B is a member of the human ovarian tumor (OTU) domain-containing deubiquitinase (DUB) family and is specific for Lys48-linked ubiquitin chains [5,6,7]. Increasing evidence indicates that OTUD7B plays a pivotal role in regulating inflammatory responses, NF-κB signaling, T-cell activation, and epidermal growth factor receptor (EGFR) trafficking [8,9]. In our previous report [10], OTUD7B expression was markedly elevated in paclitaxel-resistant MDA-MB-436 TNBC cells following treatment and was strongly positively correlated with paclitaxel IC_50_ values across TNBC cell lines. In contrast, its expression was significantly reduced in paclitaxel-sensitive HCC38 cells under the same conditions. Mechanistically, the loss of miR-1180 expression and the suppression of inflammation-related signaling pathways may contribute to OTUD7B-associated paclitaxel resistance in TNBC. However, further studies are needed to substantiate the oncogenic role of OTUD7B in driving metastatic progression.

As a result, this study aimed to explore the role of OTUD7B-mediated protein deubiquitination in promoting the metastatic progression of TNBC. Our data showed that OTUD7B prevents the destruction of Caspase 8-RIPK1 complex via its deubiquitinase activity, thereby fostering the NEMO complex-mediated activation of NF-κB and enhancing the metastatic potentials of TNBCs. These findings provide a new mechanism for TNBC metastasis but also offer a novel therapeutic strategy of combating metastatic TNBC by targeting OTUD7B activity.

## 2 Material and Methods

### 2.1 Transcriptional/Proteomic Profiles and TNBC Patient Samples

Transcriptomic data (Illumina HiSeq) for OTUD7B and proteomic profiles obtained by reverse-phase protein array (RPPA) for phosphorylated NF-κB and cleaved caspase-8 were retrieved from The Cancer Genome Atlas (TCGA) breast cancer dataset via the UCSC Xena platform (http://xena.ucsc.edu/welcome-to-ucsc-xena/). The prognostic relevance of OTUD7B expression in TNBC patients was evaluated using the Kaplan–Meier (K-M) Plotter tool (https://kmplot.com/analysis/index.php?p=service&cancer=breast). Patients were stratified into low- and high-OTUD7B expression groups on the basis of the optimal cutoff corresponding to the maximum risk separation (minimum *p*-value criterion) in the survival analysis. TNBC in the TCGA cohort was defined based on IHC-determined ER, PR, and HER2 status. In contrast, TNBC classification in the K-M Plotter dataset was based on IHC-derived ER and PR status, together with HER2 status inferred from ERBB2 gene expression using microarray data. A total of 123 TNBC patients from TCGA were included for overall survival (OS) and progression-free survival (PFS) analyses. However, only 119 patients were available for disease-specific survival (DSS) analysis due to missing survival data. In contrast, the TNBC cohort size in the K-M Plotter varied depending on the survival endpoint analyzed, including 144 patients for OS, 534 for relapse-free survival (RFS), and 424 for distant metastasis-free survival (DMFS).

### 2.2 Cell Lines and Cell Culture Conditions

The human TNBC cell lines HCC1937, HCC1806, Hs578T and MDA-MB-231, obtained from the American Type Culture Collection (ATCC, Manassas, VA, USA), were cultured in RPMI-1640 (for HCC1937 and HCC1806), Dulbecco’s modified Eagle’s medium (DMEM, for Hs578T) and Leibovitz’s (L-15) medium (for MDA-MB-231) (Gibco Life Technologies, Grand Island, NY, USA). All media were supplemented with 10% fetal bovine serum (FBS). All cells were maintained at 37°C in a humidified 5% CO_2_ incubator, except for MDA-MB-231 cells, which were cultured at 37°C under atmospheric air in a free gas exchange condition. Cell line authentication was routinely verified by short tandem repeat (STR) profiling, along with assessments of morphological features, growth characteristics, and confirmed to be free of mycoplasma contamination.

### 2.3 Plasmid Construction and Site-Directed Mutagenesis

The coding sequence of OTUD7B was amplified from human cDNA (Invitrogen, Carlsbad, CA, USA) by standard polymerase chain reaction (PCR) using primer pairs containing NheI and EcoRI restriction sites (italicized; forward-5′-TATT*GCTAGC*ATGACCCTGGACATGGATGCTGTT-3′ and reverse-5′-GG*GAATTC*TCAGAACCTGTGCACCAGGAGCTC-3′). The amplified fragment was subsequently subcloned and inserted into the NheI/EcoRI sites of the lentiviral shuttle vector pLAS/3w, which contained a puromycin resistance cassette. The resulting constructs were packaged into lentiviral particles in collaboration with the RNAi Core Facility at Academia Sinica (Taiwan). For site-directed mutagenesis, the pLAS/3w vector harboring wild-type OTUD7B was used as the template for PCR amplification with specific primer pairs (N193L: forward-5′-CTGCATGCAGGAGGCAGAGCCCATCTCCAGTAGTTG-3′ and reverse-5′-CAACTACTGGAGATGGGCTCTGCCTCCTGCATGCAG-3′; N193M: forward-5′-TGCAGGAGGCACATCCCATCTCCAGTAGTTGCCA-3′ and reverse-5′-TGGCAACTACTGGAGATGGGATGTGCCTCCTGCA-3′) using a *pfu* polymerase kit (Stratagene, La Jolla, CA, USA). The underlined nucleotides in the primer sequences indicate the sites targeted for site-directed mutagenesis. All the constructs were verified by double-stranded DNA sequencing to confirm sequence integrity.

### 2.4 Lentivirus-Driven OTUD7B shRNAs and Cell Infection

Lentiviral particles carrying nonsilencing (NS) control oligonucleotides (sequence: CCGGACACTCGAGCACTTTTTG) or OTUD7B-targeting shRNA constructs (sh1: TTGAAGAGTTTCACGTCTTTG; sh2: TGGAAATGCTCACGGTTTATA), cloned and inserted into the pLKO shuttle vector containing a puromycin resistance gene, were obtained from the RNAi Core Facility at Academia Sinica (Taiwan). TNBC cells at approximately 50% confluence in 6-well plates were pretreated with fresh medium supplemented with 5 μg/mL polybrene (Santa Cruz Biotechnology, Dallas, TX, USA) before lentiviral transduction. Cells were infected overnight with lentiviral particles encoding pLAS/3w empty vector, pLAS/3w carrying wild-type or mutant OTUD7B, pLKO vector with NS control, or pLKO vector expressing two independent OTUD7B shRNA constructs at a multiplicity of infection (MOI) of 2–10. Stable cell lines were generated using puromycin selection (10 μg/mL). Protein lysates from puromycin-resistant cells were subsequently subjected to Western blot analysis to confirm OTUD7B overexpression (OE) in HCC1937/HCC1806 cells and knockdown (KD) efficiency in Hs578T/MDA-MB-231 cells.

### 2.5 Cellular Migration Assay

Cell migration was evaluated using Boyden chambers (Neuro Probe, Inc., Gaithersburg, MD, USA) according to the protocol described in our previous study [11]. Briefly, polycarbonate membranes (8 μm pore size, 25 × 80 mm^2^) were coated on the lower surface with 10 μg of human fibronectin (Sigma, St. Louis, MO, USA) and placed over the lower chamber containing 32 μL of conditioned medium with or without the indicated inhibitors at the designated concentrations. Cells (1.5 × 10^4^) were pretreated with the indicated inhibitors for 24 h, suspended in 50 μL serum-free medium containing the corresponding inhibitor concentrations, and then seeded into the upper chamber. After incubation for 8 h (Hs578T/MDA-MB-231 cell variants) or 12 h (HCC1937/HCC1806 cell variants) at 37°C, nonmigrated cells on the upper surface were carefully removed. The cells that had migrated to the underside of the membrane were fixed with 100% methanol and stained with 10% Giemsa solution (Merck, Munich, Germany) for 1 h. The number of migrated cells was quantified under a light microscope (BX53, Olympus Corporation, Tokyo, Japan) at 400× magnification by counting ten randomly selected fields per well.

### 2.6 Determination of Protein Ubiquitination

A human ubiquitin array kit was purchased from R&D Systems^®^, Inc. (Minneapolis, MN, USA), and the array experiment was performed by Union Biomed (Taipei, Taiwan). NS control and OTUD7B-KD Hs578T cells were pretreated with the proteasome inhibitor MG132 (10 μM) for 6 h prior to protein extraction. Total proteins were extracted using Lysis Buffer 6 supplemented with protease inhibitors (#04693116001, Merck KGaA, Darmstadt, Germany) as provided in the kit. A total of 300 μg of protein lysate per sample was applied to each array membrane, and the levels of ubiquitinated proteins were measured according to the manufacturer’s protocol.

### 2.7 Cycloheximide-Chase Assay

The non-silencing (NS) control and OTUD7B-KD Hs578T cells were treated with or without cycloheximide (10 μM), a protein synthesis inhibitor, for the designated time intervals (0, 2, 4, 8, and 12 h). At the end of the incubation period, total proteins were extracted and subjected to Western blot analysis to determine the protein levels of NEMO.

### 2.8 Measurement of Caspase-8 Activity

Caspase-8 activity was detected in vector control/OTUD7B-OE HCC1937 cells and NS control/OTUD7B-KD Hs578T cells using an Elabscience^®^ Caspase-8 Activity Assay Kit (Elabscience, Houston, TX, USA) according to the manufacturer’s protocol. The collected cell pellets were resuspended in pre-chilled cell lysis buffer. (#E-CK-A38A, provided in the kit), incubated on ice for 30 min, and centrifuged at 12,000× *g* for 15 min at 4°C. The supernatants were collected and kept on ice for subsequent analysis. Protein concentrations were measured using the Bradford assay. (#5000006, Bio-Rad, Hercules, CA, USA). The pNA standard curve and sample reaction mixtures were prepared according to the manufacturer’s instructions. Samples were incubated at 37°C for 2 h, and absorbance was measured at 405 nm. Caspase-8 activity was calculated based on the pNA standard curve and expressed as U/mg protein, where one unit (U) was defined as the amount of enzyme required to cleave 1.0 nmol of the substrate Ac-IETD-pNA per hour.

### 2.9 Luciferase-Based Reporter Assay

Cells were seeded in 12-well plates and allowed to reach approximately 70% confluence before transient transfection with the pGL4.32[luc2P/NF-κB-RE/Hygro] reporter plasmid (0.25 μg; Promega, Madison, WI, USA) using Lipofectamine 3000 (Invitrogen) according to the manufacturer’s protocol. The p4.74-RLuc vector (0.0125 μg; Promega) was cotransfected as an internal control to normalize the transfection efficiency. After 24 h, the cells were lysed, and luciferase activity was measured using the Dual-Glo^®^ Luciferase Assay System (Promega). Firefly luciferase activity was first recorded, followed by quenching and subsequent detection of Renilla luciferase activity upon the addition of Dual-Glo^®^ Stop & Glo^®^ Reagent. Luminescence signals were quantified using a luminometer (Packard LumiCount™ BL 10001).

### 2.10 Western Blot Analysis

Equal amounts of total protein (20–100 μg) from the indicated samples were separated by SDS-PAGE and transferred onto polyvinylidene difluoride (PVDF) membranes. The membranes were blocked with 5% bovine serum albumin (BSA) or 5% nonfat milk in Tris-buffered saline containing 0.1% Tween-20 (TBST) for 2 h at room temperature to minimize nonspecific binding. Following blocking, the membranes were incubated overnight at 4°C with primary antibodies against OTUD7B (#16605-I-AP, 1:1000, Proteintech, Rosemont, IL, USA), phospho-NF-κB (Ser536) (#3033S, 1:1000, Cell Signaling Technology, Danvers, MA, USA), total NF-κB (#6956S, 1:1000, Cell Signaling Technology), NEMO (IKKγ, #A0917, 1:1000, ABclonal, New Taipei City, Taiwan), GAPDH (#2118S, 1:50,000, Cell Signaling Technology), GFP (#2956, 1:1000, Cell Signaling Technology), HA (#GTX115044, 1:1000, GeneTex, Irvine, CA, USA), Lamin B1 (#13435, 1:1000, Cell Signaling Technology), phospho-IκBα-S36 (#AP0999, 1:1000, ABclonal), IκBα (#9242, 1:1000, Cell Signaling Technology), phospho-IKKα/β-S176/180 (#AP0546, 1:1000, ABclonal), IKKβ (#A22425, 1:1000, ABclonal), caspase-8 (#A0215, 1:1000, ABclonal), phospho-RIP3 (Ser227) (#93654, 1:1000, Cell Signaling Technology), RIP3 (#10188, 1:1000, Cell Signaling Technology), phospho-MLKL (Ser358) (#AF7420, 1:1000, Affinity Biosciences, Cincinnati, OH, USA), and MLKL (#sc-293201, 1:1000, Santa Cruz, Dallas, TX, USA). After thorough washing, the membranes were incubated with appropriate horseradish peroxidase (HRP)-conjugated secondary antibodies (1:10,000) for 1 h at room temperature. Protein signals were visualized using an enhanced chemiluminescence (ECL) detection system (Amersham Biosciences, GE Healthcare, Billerica, MA, USA).

### 2.11 Annexin V/7-AAD Staining and Flow-Cytometric Analysis

Collected TNBC cells were resuspended in Annexin V binding buffer (BioLegend, San Diego, CA, USA) to a final concentration of 1 × 10^7^ cells/mL. A total of 100 μL of the cell suspension was transferred to a 5-mL tube and incubated with 5 μL of FITC-conjugated Annexin V (BioLegend) and 5 μL of 7-Aminoactinomycin D (7-AAD; BioLegend). Following a 15-min incubation at room temperature, 400 μL of Annexin V binding buffer was added, and the samples were immediately analyzed by flow cytometry (Attune NxT Flow Cytometer; Thermo Fisher Scientific, Waltham, MA, USA).

### 2.12 Immunohistochemistry Staining

Deparaffinized TNBC tumor tissues (*n* = 20) obtained from Cardinal Tien Hospital (CTH), in accordance with CTH Institutional Review Board approval (CTH-112-3-1-035) and the Declaration of Helsinki, as well as lung tissues derived from mice transplanted with NS control or OTUD7B-KD Hs578T cells, were subjected to antigen retrieval using 10 mM citrate buffer (pH 6.0), followed by quenching of endogenous peroxidase activity with 3% hydrogen peroxide. Written informed consent was obtained from all participants, and TNBC tissue sections were prepared by CTH pathologists. Mouse lung tissues were fixed in 10% neutral phosphate-buffered formalin (Bioman Scientific, New Taipei City, Taiwan) and subsequently processed for paraffin embedding. Tissue sections were prepared by pathologists at Taipei Medical University. To minimize nonspecific binding, the sections were blocked with normal goat serum (1:20) for 1 h and subsequently incubated overnight at 4°C in a humidified chamber with primary antibodies against cleaved Caspase-8 (1:500; GTX86893; GeneTex, Hsinchu, Taiwan) or phospho-NF-κB (1:150; CST #4808; Cell Signaling Technology). After extensive washing, the sections were incubated with 100 μL of HRP-conjugated secondary antibody [N-Histofine^®^ Simple Stain MAX PO (MULTI), Nichirei Biosciences, Tokyo, Japan]) for 1 h. Immunoreactive signals were developed using a DAB substrate kit (PolyDetector Liquid DAB HRP Brown Kit). Finally, the sections were counterstained with hematoxylin, dehydrated, cleared in xylene, mounted, and examined under a light microscope (BX53, Olympus Corporation).

### 2.13 Animal Experiment

Advanced Severe Immunodeficiency (ASID) B6.129S4-Il2rgtm1Wjl/J mice (6–8 weeks old, female) were obtained from the National Laboratory Animal Center (Taipei, Taiwan) and maintained under specific pathogen-free conditions in accordance with institutional guidelines. The animal studies were performed following the guidelines and protocols approved by the Institutional Animal Care and Use Committee (IACUC) of Taipei Medical University (approval no. LAC-2020-0295). All procedures were conducted and reported in accordance with the ARRIVE Essential 10 guidelines (https://arriveguidelines.org/resources/author-checklists). For the lung colonization model, mice (*n* = 6 per group) were injected via the tail vein with 1 × 10^6^ luciferase-expressing NS or OTUD7B-KD Hs578T cells suspended in 100 μL PBS, as previously described [12]. Lung colonization was monitored weekly by measuring bioluminescent signals using an *in vivo* imaging system (IVIS). At the experimental endpoint, the mice were humanely euthanized by CO_2_ inhalation followed by cervical dislocation according to institutional-approved protocols, and lung tissues were collected for subsequent histological analysis.

### 2.14 Pathway Enrichment Analysis

Differentially expressed genes (DEGs) analysis was performed by comparing TCGA RNA sequencing data of TNBC patients without or with lymph node metastasis. The data were obtained from the UCSC Xena platform (http://xena.ucsc.edu/welcome-to-ucsc-xena/). and analyzed using the limma-voom algorithm. DEGs were defined as genes with an absolute log_2_ fold change (|log_2_FC|) ≥ 1.5, *p* < 0.01, and a false discovery rate (FDR) < 0.05. Pathway enrichment analysis against the DEGs was further performed using Enrichr, an interactive and collaborative HTML5 gene list enrichment analysis tool, with pathway annotations derived from the Reactome database. 

### 2.15 Statistical Analysis

Statistical analyses were performed using GraphPad Prism 7.0 (GraphPad Software, Inc., San Diego, CA, USA). Data from at least three independent experiments are presented as the mean ± standard deviation (SD). The Mann–Whitney U test was used to compare two independent groups, whereas the Friedman test was used to compare three or more related groups in cell-based and animal experiments. Differences were considered statistically significant when the *p*-value was < 0.05.

## 3 Results

### 3.1 Elevated OTUD7B Expression Is Correlated with Unfavorable Clinical Outcomes in TNBC Patients

**Figure 1:**
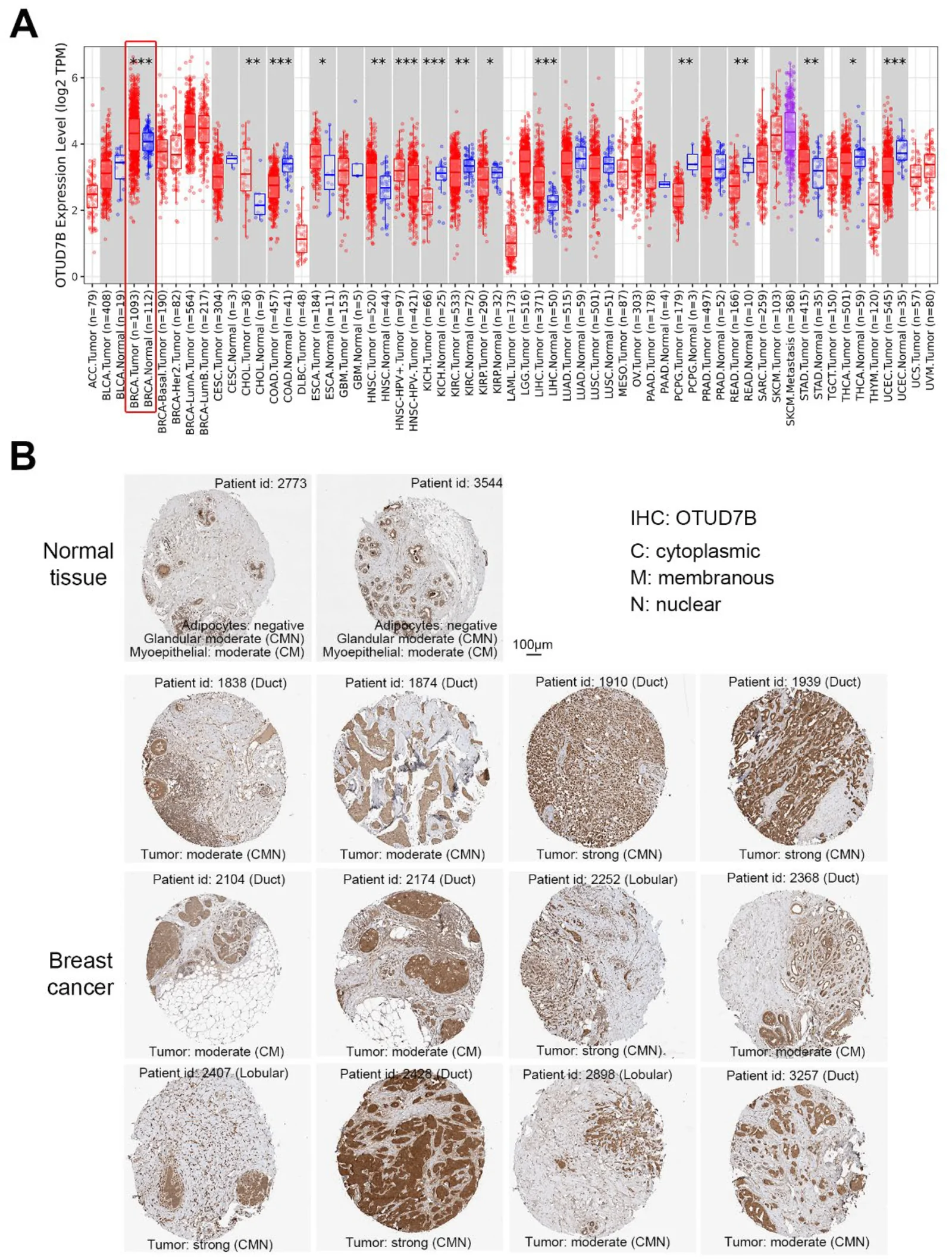
**OTUD7B is upregulated in primary tumors compared to normal mammary tissues from breast cancer patients.** (**A**) Boxplot for the transcriptional profile of OTUD7B in the primary tumors (red) and adjacent normal tissues (blue) derived from various cancer types, including breast cancer (BRCA), shown in red box, deposited in the TCGA database. *p < 0.05, **p < 0.01, and ***p < 0.001. (**B**) Immunohistochemistry staining for OTUD7B protein levels in the normal mammary tissues and breast cancer tissues from the Human Protein Atlas database. Abbreviations: ACC, Adrenocortical carcinoma; BLCA, Bladder urothelial carcinoma; BRCA, Breast invasive carcinoma; CESC, Cervical and endocervical cancers; CHOL, Cholangiocarcinoma; COAD, Colon adenocarcinoma; DLBC, Lymphoid Neoplasm Diffuse Large B-cell Lymphoma; ESCA, Esophageal carcinoma; GBM, Glioblastoma multiforme; HNSC, Head and Neck squamous cell carcinoma; KICH, Kidney Chromophobe; KIRC, Kidney renal clear cell carcinoma; KIRP, Kidney renal papillary cell carcinoma; LAML, Acute Myeloid Leukemia; LGG, Brain Lower Grade Glioma; LIHC, Liver hepatocellular carcinoma; LUAD, Lung adenocarcinoma; LUSC, Lung squamous cell carcinoma; MESO, Mesothelioma; OV, Ovarian serous cystadenocarcinoma; PAAD, Pancreatic adenocarcinoma; PCPG, Pheochromocytoma and Paraganglioma; PRAD, Prostate adenocarcinoma; READ, Rectum adenocarcinoma; SARC, Sarcoma; SKCM, Skin Cutaneous Melanoma; STAD, Stomach adenocarcinoma; TGCT, Testicular Germ Cell Tumors; THCA, Thyroid carcinoma; THYM: Thymoma; UCEC, Uterine Corpus Endometrial Carcinoma; UCS, Uterine Carcinosarcoma; UVM, Uveal Melanoma; IHC, immunohistochemistry.

**Figure 2:**
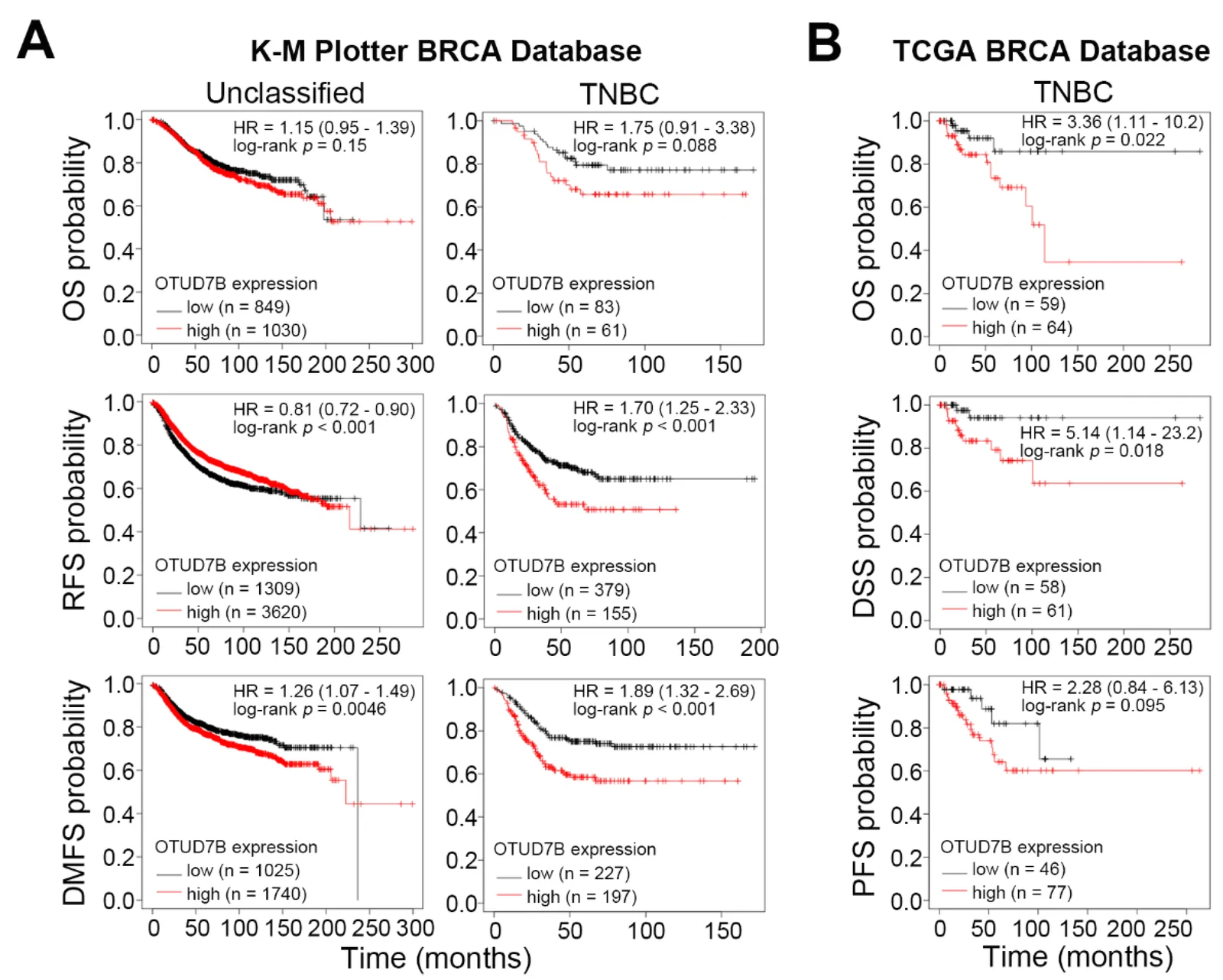
**OTUD7B upregulation correlates with a poorer prognosis in triple-negative breast cancer (TNBC).** (**A**,**B**) Kaplan-Meier analyses for OTUD7B mRNA levels using overall survival (OS), relapse-free survival (RFS), and distant metastasis-free survival (DMFS) probability against unclassified and TNBC patients deposited in the K-M Plotter database (**A**) and OS, disease-specific survival (DSS), and progression-free survival (PFS) probability against TCGA TNBC patients (**B**). The patients were stratified into the low- and high-OTUD7B expression under a maximal risk condition of Kaplan-Meier survival analysis. HR: hazard ratio.

The transcription profiling revealed that OTUD7B mRNA levels in the primary tumors are higher than those of normal adjacent tissues from breast cancer patients (Fig. 1A). Similar views were also found in other cancer types, such as head and neck squamous carcinoma (Fig. 1A). Accordingly, immunohistochemistry staining results showed that the protein levels of OTUD7B in breast cancer tissues are higher than those of normal breast tissues (Fig. 1B). Compared to the unclassified breast cancers, Kaplan-Meier analyses demonstrated that an increased level of OTUD7B highly correlates with a poorer overall, relapse-free, and distant metastasis-free survival rate in TNBC patients deposited in the K-M Plotter website (Fig. 2A). We next used the TNBC cohort from the TCGA database to validate these findings. The data also showed that a higher OTUD7B expression refers to a poorer overall, disease-specific, and progression-free survival rate in TNBC patients (Fig. 2B). Although OTUD7B expression was significantly associated with overall survival in univariate analysis, this association was attenuated and no longer statistically significant after multivariate adjustment (Table A1). This finding suggests that the observed effect may be influenced by other established clinical variables rather than representing an independent prognostic factor.

### 3.2 OTUD7B Expression Causally Associates with the Metastatic Potentials of TNBC Cells

**Figure 3:**
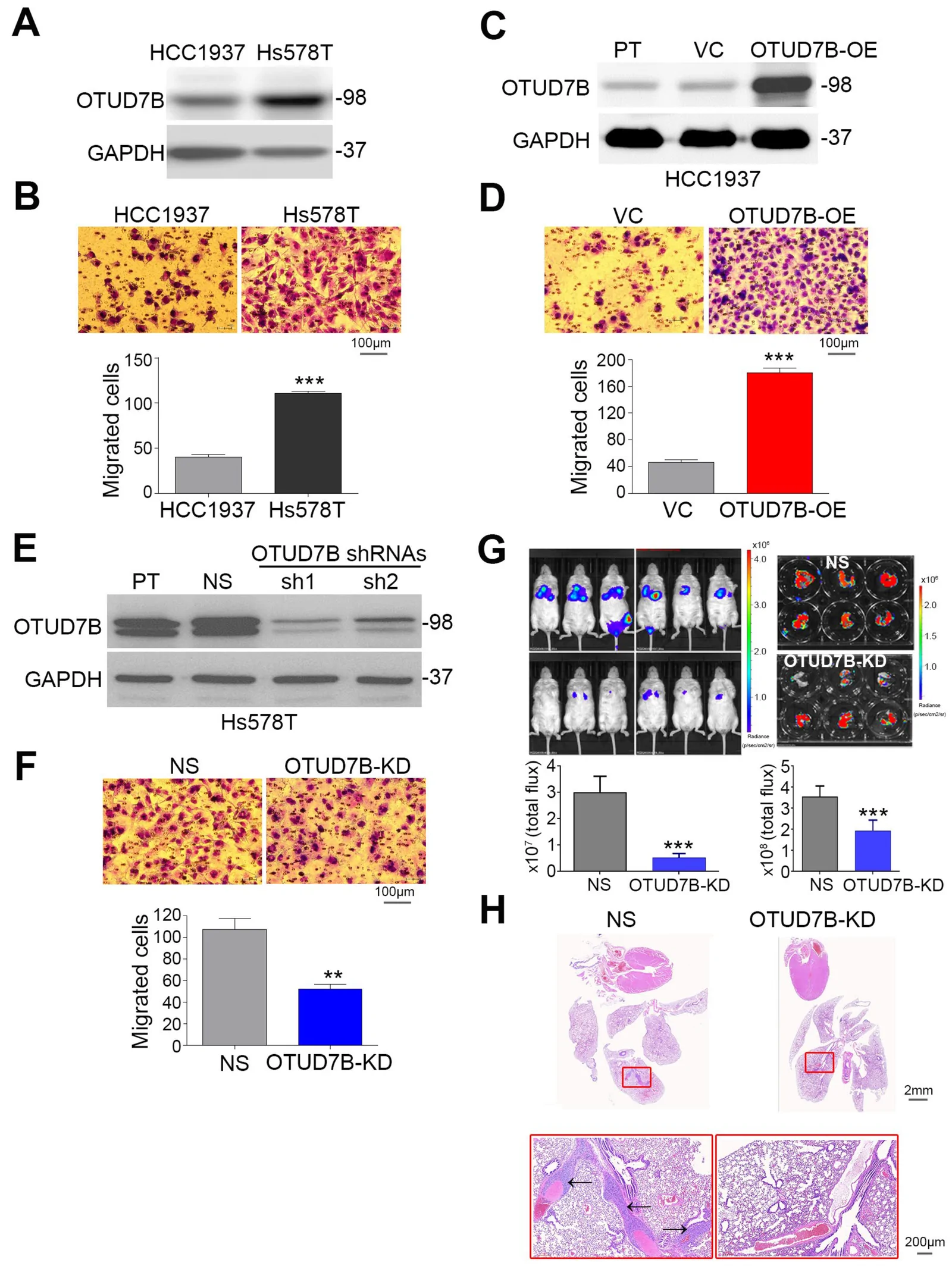
**OTUD7B expression causally associates with the metastatic potentials of triple-negative breast cancer (TNBC) cells.** (**A**–**F**) Western blot analyses for OTUD7B protein levels (**A**,**C**,**E**) and Giemsa staining (upper)/histogram (lower) for migrated cells (**B**,**D**,**F**) in the HCC1937/Hs578T cells (**A**,**B**), parental (PT)/vector control (VC)/OTUD7B-OE HCC1937 cells (**C**,**D**) and PT/non-silencing (NS) control/OTUD7B-KD Hs578T cells (**E**,**F**). In (**F**), OTUD7B-KD cells were generated by the OTUD7B sh1-shRNA clone. **p < 0.01 and ***p < 0.001. In panels (**A**,**C**,**E**), GAPDH was used as an internal control of protein loading. (**G**,**H**) Luminescent intensity captured by IVIS image system (**G**, upper panel) and quantified as total photon flux, presented as histograms (**G**, lower panel) in tumor-bearing mice transplanted with the NS and OTUD7B-KD Hs578T cells for 6 weeks (left)/lungs from tumor-bearing mice (right) at the end of the experiment and hematoxylin/eosin staining (H) for lung tissues from the representative tumor-bearing mice. ***p < 0.001. The arrows indicate the tumor colonies of Hs578T cells.

**Figure A1:**
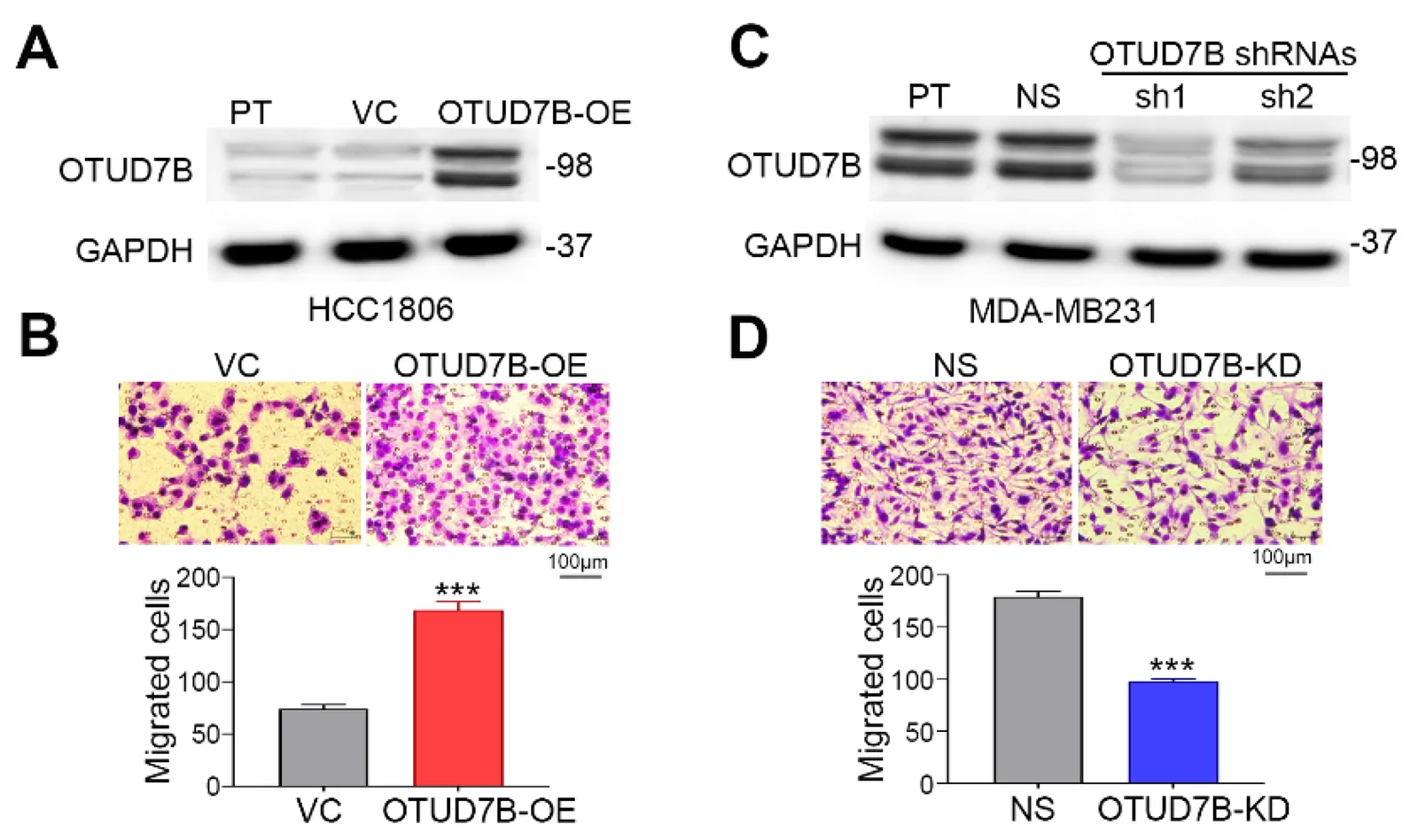
**OTUD7B expression is causally associated with the cellular migration ability in triple-negative breast cancer (TNBC) cells.** (**A**–**D**) Western blot analysis of OTUD7B protein expression (**A**,**C**) and Giemsa staining (upper panels) with quantification of migrated cells (lower panels; histograms) in parental (PT), vector control (VC), and OTUD7B-overexpressing (OE) HCC1806 cells (**A**,**B**); and PT, nonsilencing (NS) control, and OTUD7B-knockdown (KD) MDA-MB-231 cells (**C**,**D**). In (**D**), OTUD7B knockdown was achieved using an OTUD7B sh1-shRNA clone. ***p < 0.001. GAPDH served as the loading control in (**A**,**C**).

**Figure A2:**
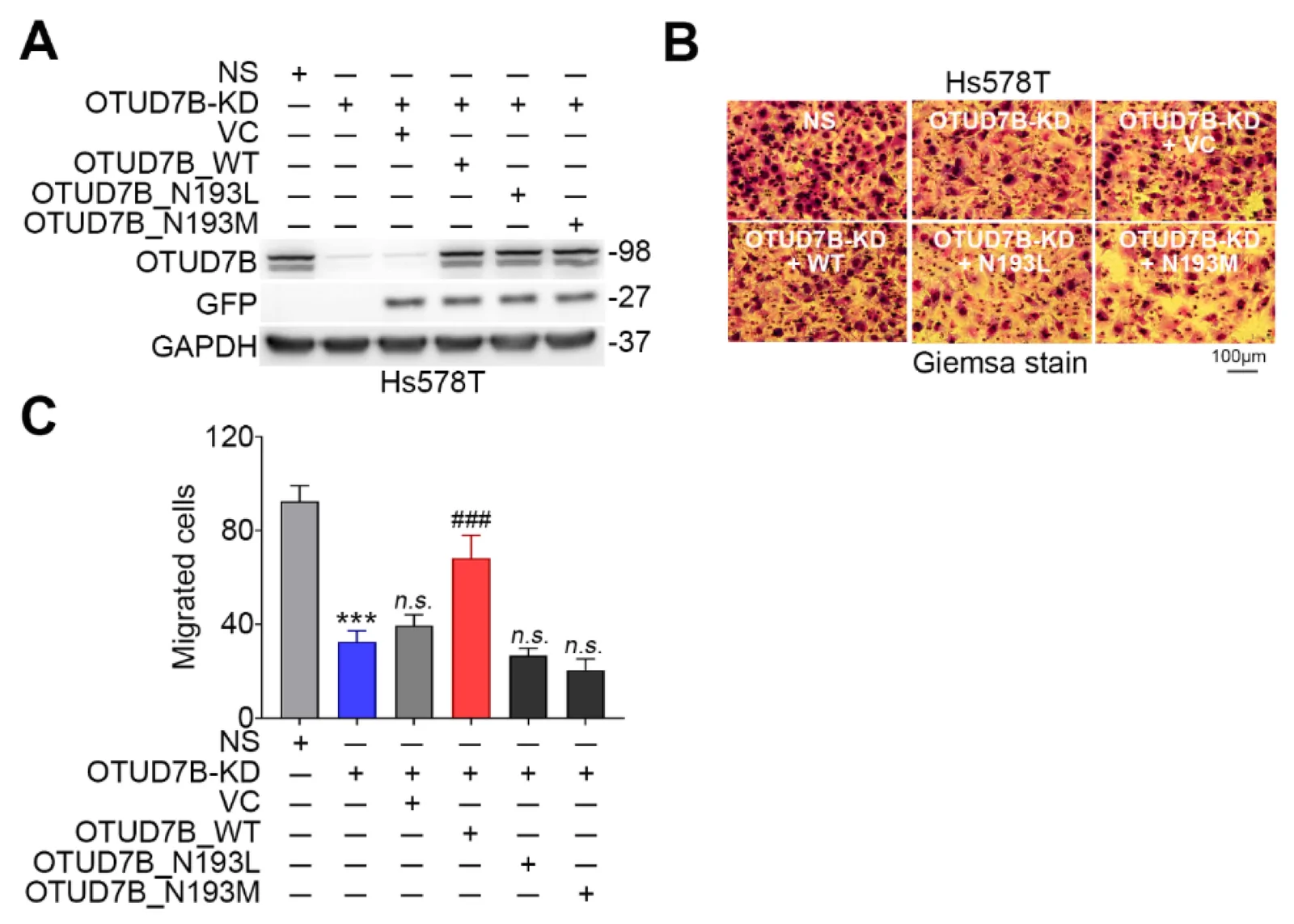
**Rescue experiments of OTUD7B in Hs578T cells.** (**A**–**C**) Western blot analysis of OTUD7B protein expression (**A**), representative images of Giemsa staining (**B**), and quantification of migrated cells (**C**) in nonsilencing (NS) control and OTUD7B-knockdown (KD) Hs578T cells transfected without (vector control, VC) or with ectopic expression of wild-type or N193L/N193M-mutant OTUD7B. Green fluorescent protein (GFP) used as control for the transient transfection efficiency of the constructs derived from pIRES2-EGFP vector, and GAPDH served as the protein loading control in (**A**). Statistical significance is indicated as ***p < 0.001 compared with the NS control group and ^###^*p* < 0.001 compared with the OTUD7B-KD group. n.s., not significant, compared with the OTUD7B-KD group.

Based on our previous reports [12], we firstly dissected the endogenous levels of OTUD7B in HCC1937 cells (poorly metastatic) and Hs578T cells (highly metastatic). The data showed that Hs578T cells, in comparison with HCC1937 cells, harbor a higher OTUD7B expression and exhibit a stronger migration (Fig. 3A,B). The enforced expression of exogenous OTUD7B gene in HCC1937 (Fig. 3C) dramatically potentiated cellular migration ability (Fig. 3D), whereas the knockdown of endogenous OTUD7B levels by its 2 specific shRNA clones in Hs578T cells (Fig. 3E) markedly mitigated cellular migration ability (Fig. 3F). The similar views were also found in the poorly migrated HCC1806 cells and highly migrated MDA-MB-231 cells (Fig. A1A–D). Re-expression of wild-type OTUD7B effectively restored the migratory ability of OTUD7B-knockdown Hs578T cells (Fig. A2A–C). Moreover, lung colony-forming assay revealed that OTUD7B knockdown predominantly suppresses the lung colonization capacity of Hs578T cells in tumor-bearing mice (Fig. 3G,H).

### 3.3 The Deubiquitinating Activity of OTUD7B Is Required to Promote TNBC Metastasis

**Figure 4:**
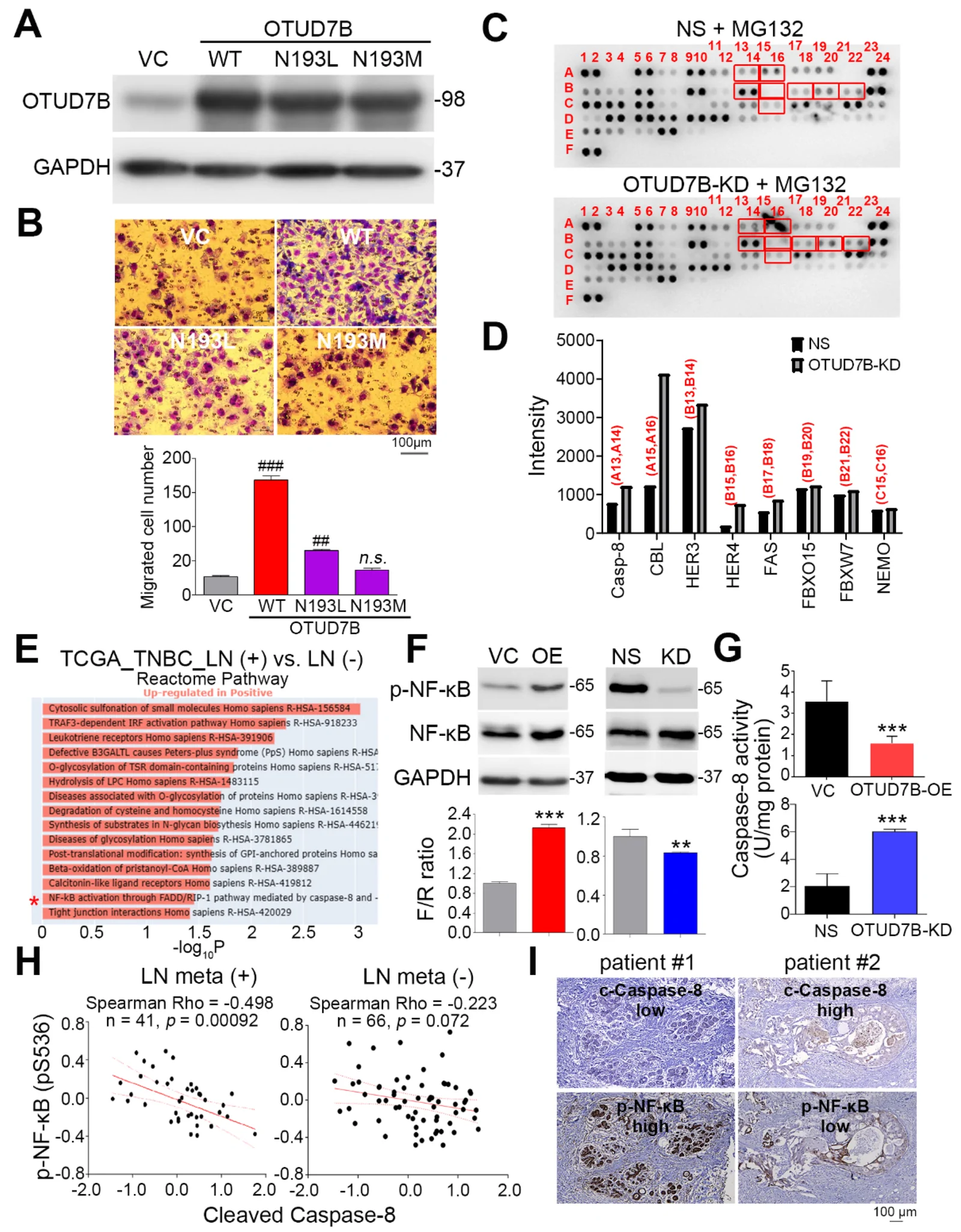
**OTUD7B deubiquitinating activity is required for promoting triple-negative breast cancer (TNBC) metastasis via Caspase-8-regulated NF-κB activation.** (**A**,**B**) Western blot analyses for OTUD7B protein levels (**A**) and Giemsa staining (upper)/histogram (lower) for migrated cells (**B**) in HCC1937 cells overexpressing vector control (VC), wild-type OTUD7B, and mutant (N193L and N193M) OTUD7B genes. n.s, not significant, ^##^*p* < 0.01 and ^###^*p* < 0.001, respectively. (**C**,**D**) Dot plot analyses for the protein ubiquitination levels of detected proteins, as shown in Fig. A3 (**C**), and a histogram for the normalized protein ubiquitination intensity of indicated proteins in the NS and OTUD7B-KD Hs578T cells. (**E**) Histogram for the statistical *p*-values (shown in −log_10_P) of Reactome Pathway gene sets in the Pathway enrichment analysis against the differentially expressed genes derived from the comparison between lymph node metastasis-positive and negative TNBC tissues deposited in the TCGA database. The symbol “*” indicates the pathway in which NF-κB activation through FADD/RIP-1 (RIPK1) pathway mediated by caspase-8 and -10. (**F**) Western blot analyses for phosphorylated NF-κB (p-NF-κB), total NF-κB, and GAPDH protein levels (upper) and histograms for the NF-κB DNA binding activity determined by luciferase reporter assay, presented as the ratio of firefly to *Renilla* luciferase activity (F/R) (lower) in the VC/OTUD7B-OE HCC1937 cells (left) and NS/OTUD7B-KD Hs578T cells (right). GAPDH was used as an internal control of protein loading. (**G**) The histograms for the Caspase-8 activity in the VC/OTUD7B-OE HCC1937 cells (upper) and NS/OTUD7B-KD Hs578T cells (lower). **p < 0.01 and ***p < 0.001, respectively. (**H**) Scatter plot for the protein levels of cleaved (active) Caspase-8 and p-NF-κB in the primary tumors derived from TCGA TNBC patients with (left) or without (right) lymph node metastasis. (**I**) Immunohistochemistry staining for the protein levels of cleaved Caspase-8 (upper) and p-NF-κB (lower) in the representative TNBC tissues from Cardinal Tien Hospital.

**Figure A3:**
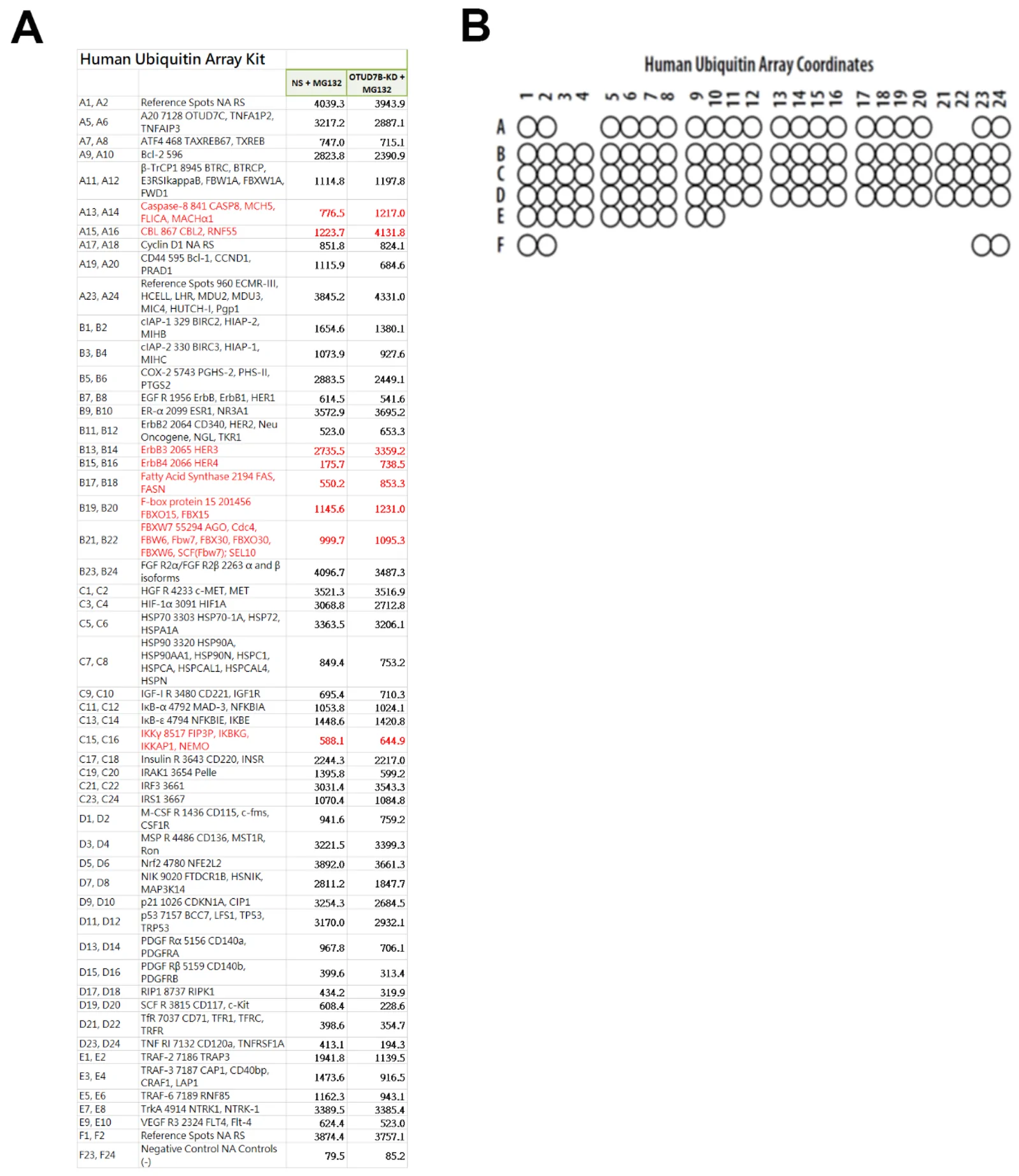
**Supplementary information for Fig. 4C,D.** (**A**) The identities of dot plots in Human Ubiquitin Array Kit and intensity of spots in the samples from the NS control and OTUD7B-KD Hs578T cells in the presence of MG132 (10 μM). (**B**) The coordinates of Human Ubiquitin Array Kit.

**Figure A4:**
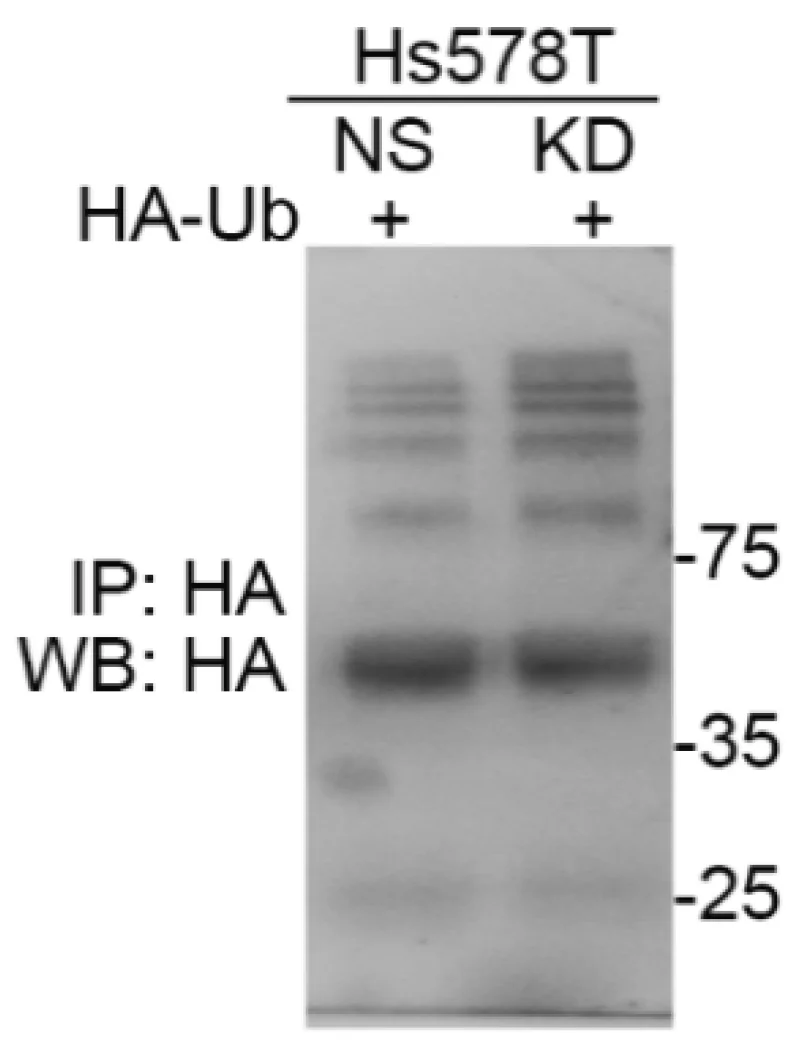
Intracellular protein ubiquitination following OTUD7B knockdown in Hs578T cells. Western blot analysis of immunoprecipitates obtained from whole cell lysates of hemagglutinin (HA)-tagged ubiquitin (HA-Ub)-transfected non-silencing (NS) control and OTUD7B-knockdown (KD) Hs578T cells using an anti-HA antibody and subsequently probed with anti-HA antibody.

To delineate if the OTUD7B deubiquitinating activity is crucial for TNBC metastasis, we next generated 2 enzyme-dead constructs, N193L and N193M, according to a previous report [13]. Compared to HCC1937 cells overexpressing wild-type OTUD7B, the enforced expression of N193L and N193M mutants slightly or did not affect the migration ability of HCC1937 cells (Fig. 4A,B). Similar views were also found in the re-expression of ectopic wild-type or N193L/N193M-mutant OTUD7B in the OTUD7B-KD Hs578T cells (Fig. A2A–C). It indicates that deubiquitinating activity is required for the OTUD7B-promoted TNBC metastasis. To identify the possible protein targets that mediate the OTUD7B-promoted TNBC metastatic progression, we next detected the changes in ubiquitinated protein levels by comparing OTUD7B-silenced Hs578T cells with non-silenced control cells using the Human Ubiquitin Array Kit. The data showed that the ubiquitination of several proteins, such as Caspase-8 and NEMO, is enhanced after OTUD7B knockdown (Fig. 4C,D and Fig. A3). Accordingly, the intracellular protein ubiquitination was also increased after OTUD7B knockdown in Hs578T cells (Fig. A4). Intriguingly, pathway enrichment analysis using the Xena website against primary tumors from TCGA TNBC patients without or with lymph node metastasis unveiled that the signaling axis in which NF-κB activation through the FADD/RIPK1 pathway mediated Caspase-8 and -10 is significantly (*p* = 0.035) upregulated in the lymph node metastatic TNBCs (Fig. 4E).

### 3.4 OTUD7B Suppresses Caspase-8 Activity via Preventing Its Ubiquitination to Activate NF-κB in TNBC Cells

**Figure A5:**
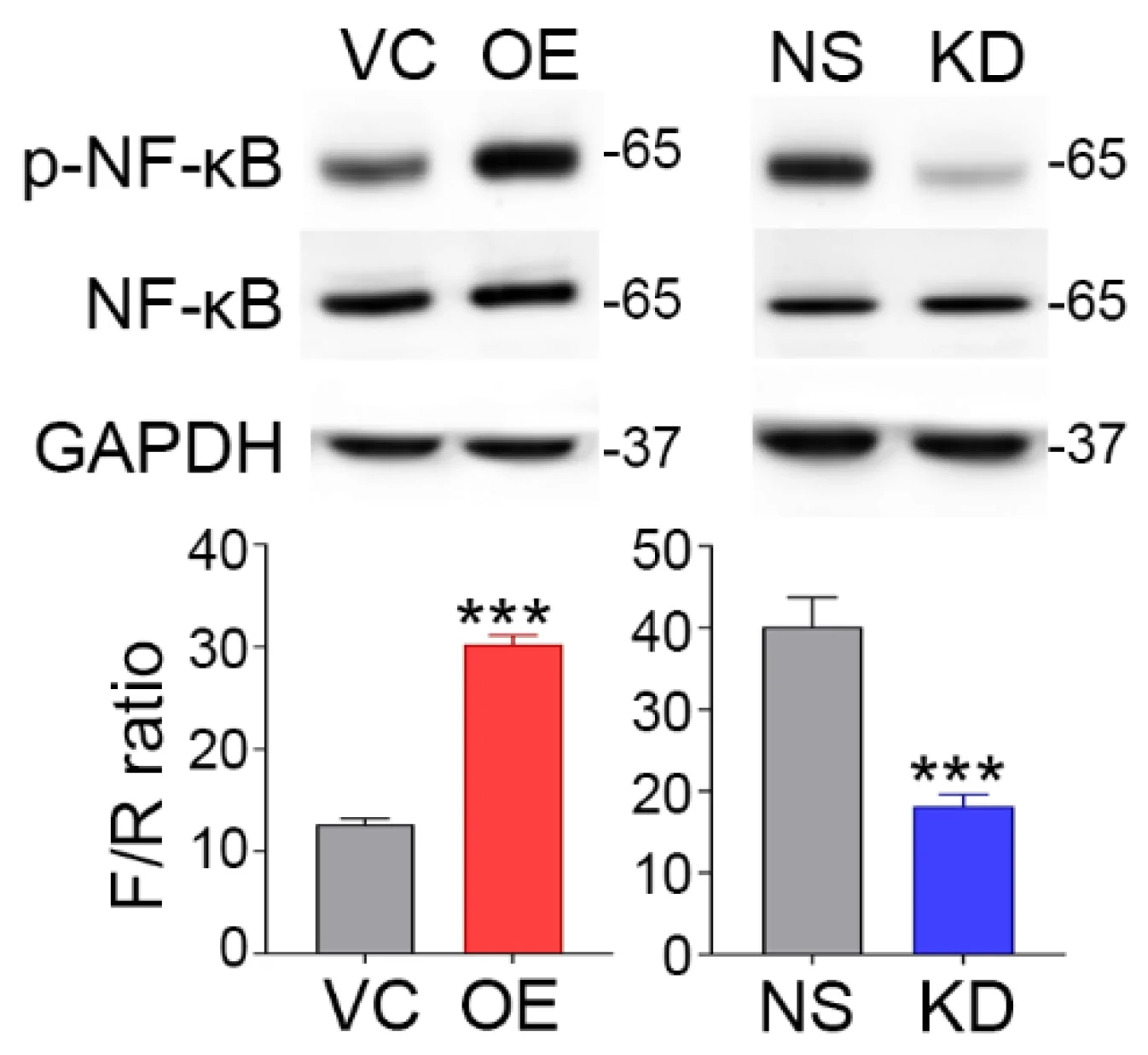
Western blot analysis of phosphorylated NF-κB (p-NF-κB), total NF-κB, and GAPDH protein levels (upper panels), along with quantification of NF-κB transcriptional activity measured by a luciferase reporter assay, presented as the ratio of firefly to *Renilla* luciferase activity (F/R) (lower panels), in vector control (VC) *versus* OTUD7B-overexpresing (OE) HCC1806 cells (left) and non-silencing (NS) control *versus* OTUD7B-knockdown (KD) MDA-MB-231 cells (right). GAPDH served as the loading control. ***p < 0.001.

**Figure A6:**
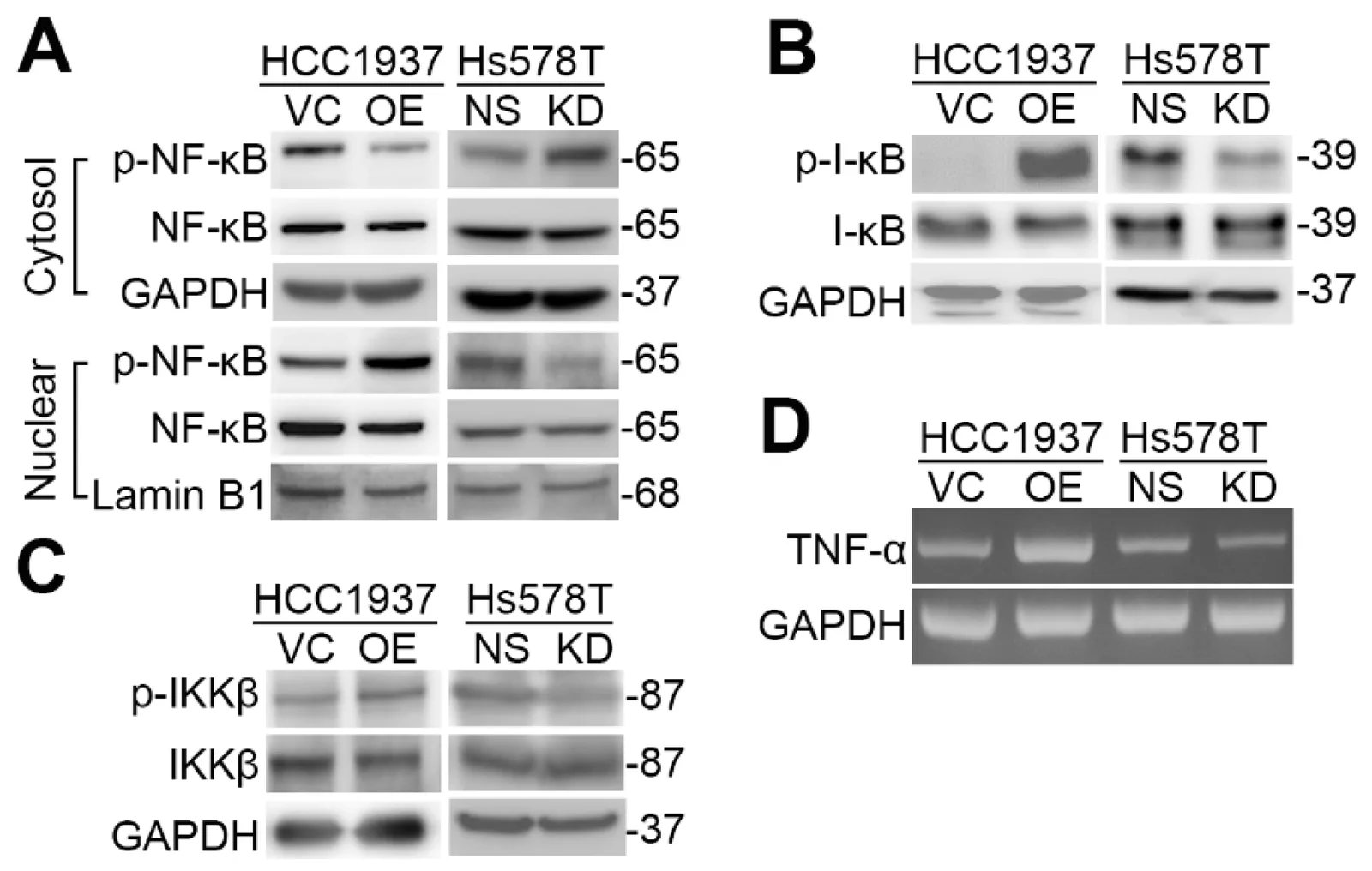
**Nuclear translocation of NF-κB, phosphorylation of IκB and IKKβ, and TNF-α expression following OTUD7B overexpression or knockdown in TNBC cells.** (**A**–**C**) Western blot analysis of phosphorylated NF-κB (p-NF-κB) and total NF-κB protein levels in cytosolic and nuclear fractions (**A**), as well as p-IκB/total IκB (**B**) and p-IKKβ/total IKKβ (**C**) expression in whole-cell lysates derived from vector control (VC) or OTUD7B-overexpressing (OTUD7B-OE) HCC1937 cells and nonsilencing (NS) control or OTUD7B-knockdown (OTUD7B-KD) Hs578T cells. GAPDH and Lamin B1 served as loading controls for the cytosolic and nuclear fractions, respectively, in (**A**). GAPDH also served as the loading control in (**B**,**C**). (**D**) RT-PCR analysis of TNF-α mRNA expression in VC/OTUD7B-OE HCC1937 cells and NS/OTUD7B-KD Hs578T cells. GAPDH served as the experimental control.

**Figure A7:**
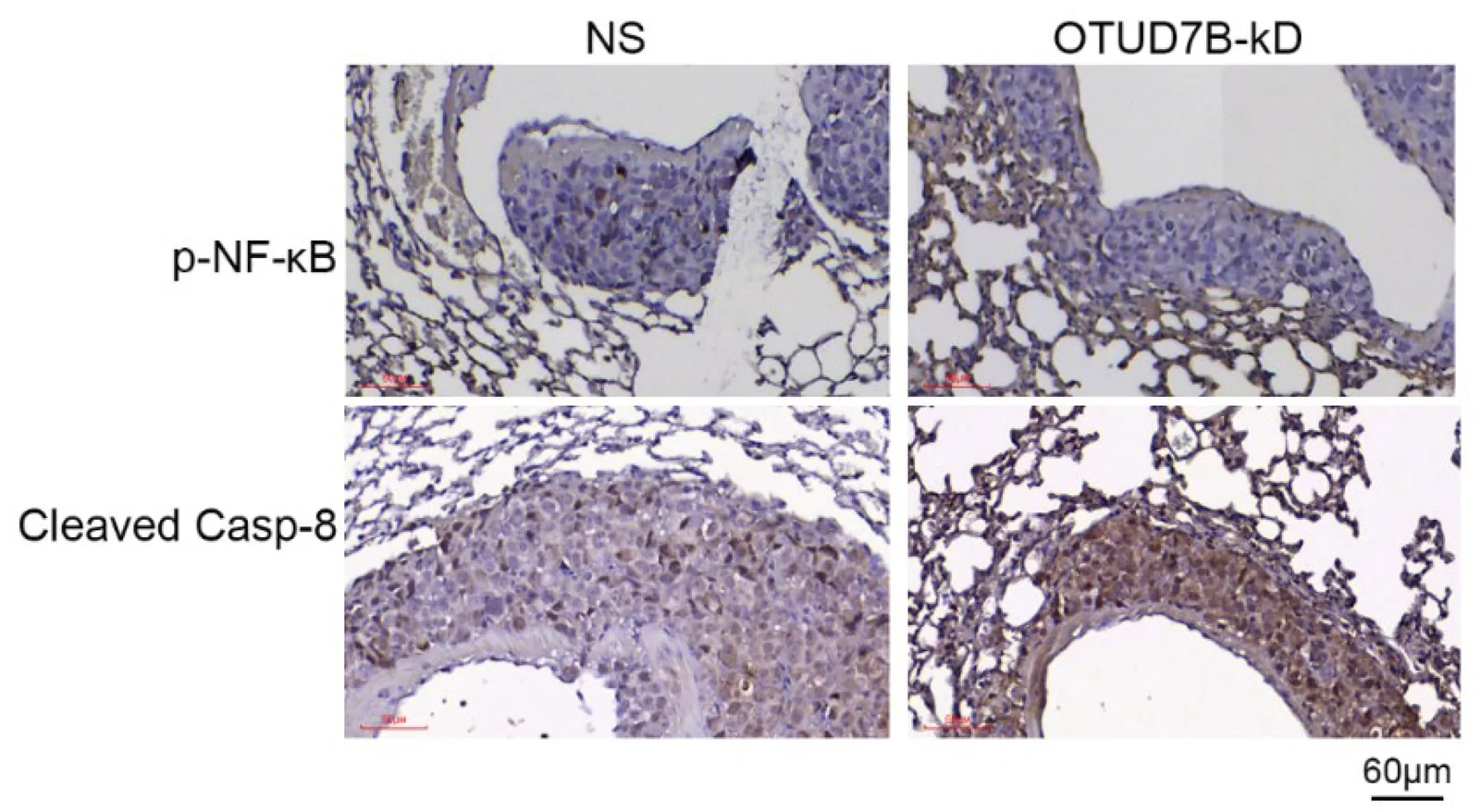
*In vivo* activity of NF-κB and Caspase-8 following OTUD7B knockdown in Hs578T cells. Immunohistochemical (IHC) staining of phosphorylated NF-κB (p-NF-κB) and cleaved (active) Caspase-8 in lung tumor colonies from tumor-bearing mice injected with nonsilencing (NS) control or OTUD7B-knockdown (OTUD7B-KD) Hs578T cells.

Our previous report has demonstrated that NF-κB activation dictates the TNBC metastasis [14]. Our data showed that OTUD7B overexpression in HCC1937 and HCC1806 cells promotes, but its knockdown in Hs578T and MDA-MB-231 cells suppresses, NF-κB activity as judged by the changes in the phosphorylated protein levels and DNA-binding activity (Fig. 4F and Fig. A5), as well as nuclear translocation of NF-κB, I-κB phosphorylation, IKKβ phosphorylation, and TNFα expression (Fig. A6A–D). Conversely, OTUD7B overexpression in HCC1937 cells reduced Caspase-8 activity; whereas its knockdown in Hs578T cells elevated Caspase-8 activity (Fig. 4G). By analyzing TCGA proteomic datasets, we found that primary tumors from TNBC patients with lymph node metastasis exhibited a significant negative correlation between phosphorylated NF-κB and cleaved Caspase-8 protein levels (*p* < 0.001), compared with tumors from patients without lymph node metastasis. (Fig. 4H). Similar views were also found in immunohistochemistry staining for phosphorylated NF-κB and cleaved Caspase-8 against TNBC tissues from Cardinal Tien Hospital (Fig. 4I) and lung tumor colonies from tumor-bearing mice injected with NS control or OTUD7B-KD Hs578T cells (Fig. A7).

### 3.5 NEMO Complex Mediates the OTUD7B-Fostered Metastatic Ability in TNBC Cells

**Figure 5:**
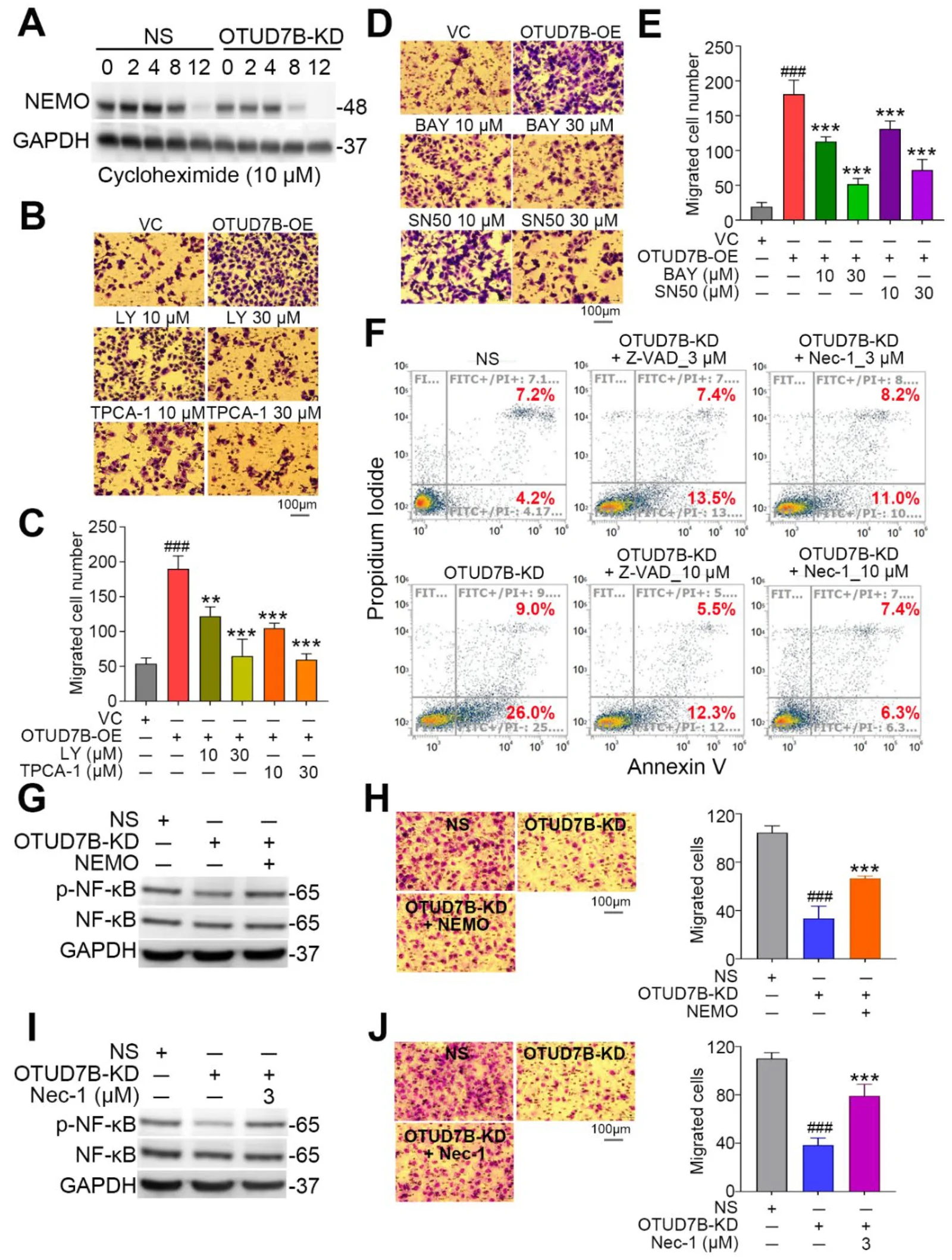
**The activity of the NEMO complex and NF-κB is required for the OTUD7B-promoted metastasis, and OTUD7B knockdown induces necroptosis in triple-negative breast cancer (TNBC) cells.** (**A**) Western blot analysis for protein levels of NEMO and GAPDH in the different time intervals of the Cycloheximide-chase assay against the NS and OTUD7B-KD Hs578T cells. GAPDH was used as an internal control of protein loading. (**B**–**E**) Giemsa staining (**B**,**D**) and histograms (**C**,**E**) for the migrated cells in the trans-well cultivation of VC and OTUD7B-OE HCC1937 cells in the absence or presence of IKKβ inhibitors LY2409881 (LY) and TPCA-1 (**B**,**C**) and NF-κB inhibitors BAY11-7082 (BAY) and SN50 (**D**,**E**) at the indicated concentrations for 16 h. (**F**) Annexin V/Propidium Iodide (PI) staining-based Flow-cytometric analyses against the NS control and OTUD7B-KD Hs578T cells in the absence or presence of pan-caspase inhibitor Z-VAD and necroptosis inhibitor (Nec-1) at the designated concentrations. ^###^*p* < 0.001 as compared to the VC group; **p < 0.01 and ***p < 0.001, as compared to the OTUD7B-OE group. (**G**–**J**) Western blot analysis of p-NF-κB, total NF-κB, and GAPDH (**G**,**I**) and Giemsa staining (left panels) with quantification of migrated cells (right panels; histograms) in NS control and OTUD7B-KD Hs578T cells without or with NEMO re-expression (**G**,**H**) or Nec-1 treatment (**I**,**J**). ^###^*p* < 0.001, as compared to the NS group; ***p < 0.001 as compared to the OTUD7B-KD group.

**Figure A8:**
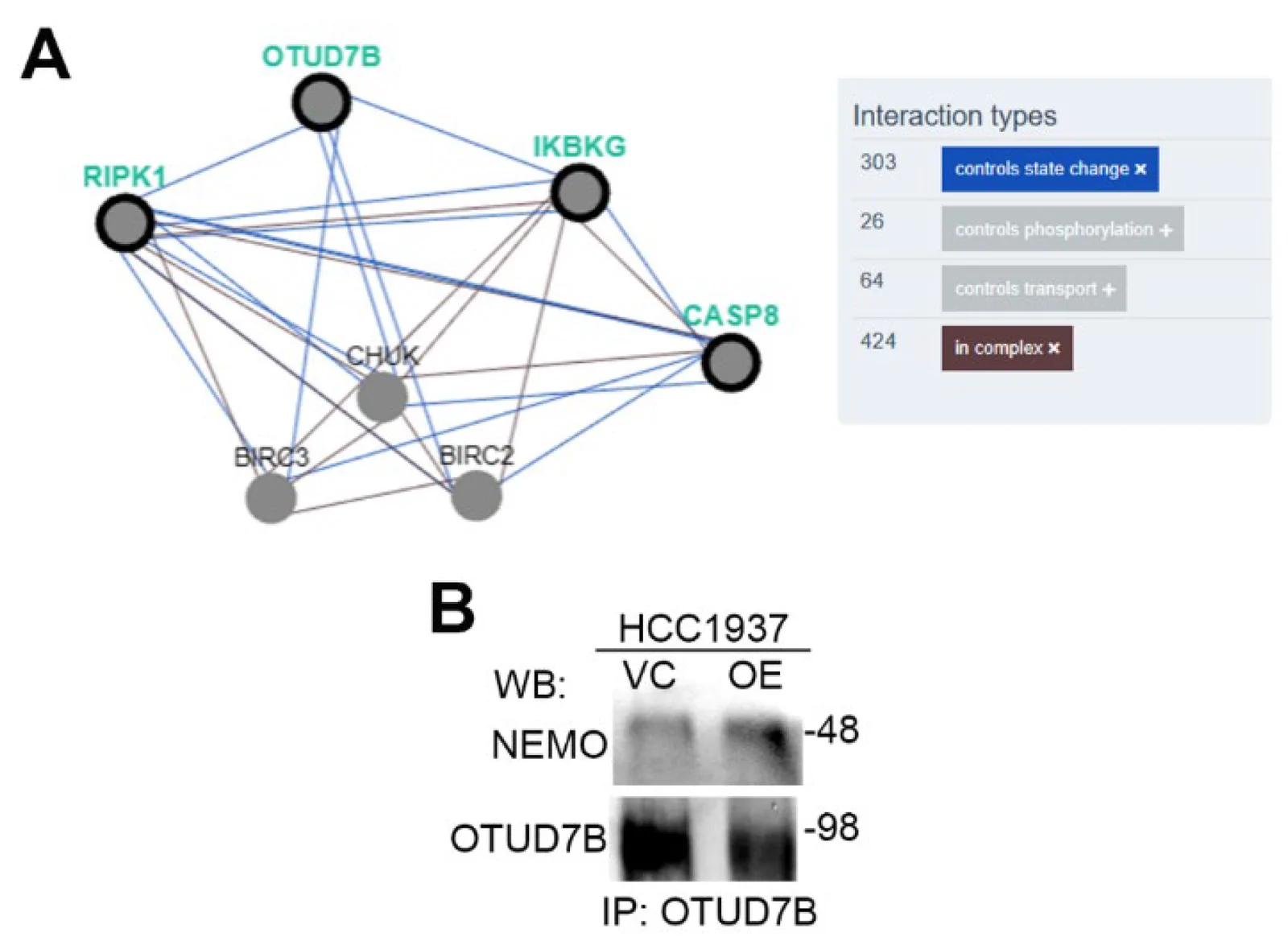
**Potential protein–protein interactions among OTUD7B, Caspase-8, NEMO, and RIPK1.** (**A**) Network analysis generated using the Pathway Commons Network Visualizer for OTUD7B, Caspase-8 (CASP8), NEMO (IKBKG), and RIPK1. Blue and brown lines indicate “controls state change of” and “in complex with” interactions, respectively. (**B**) Western blot analysis of immunoprecipitates obtained using an anti-OTUD7B antibody and subsequently detected with anti-NEMO and anti-OTUD7B antibodies.

**Figure A9:**
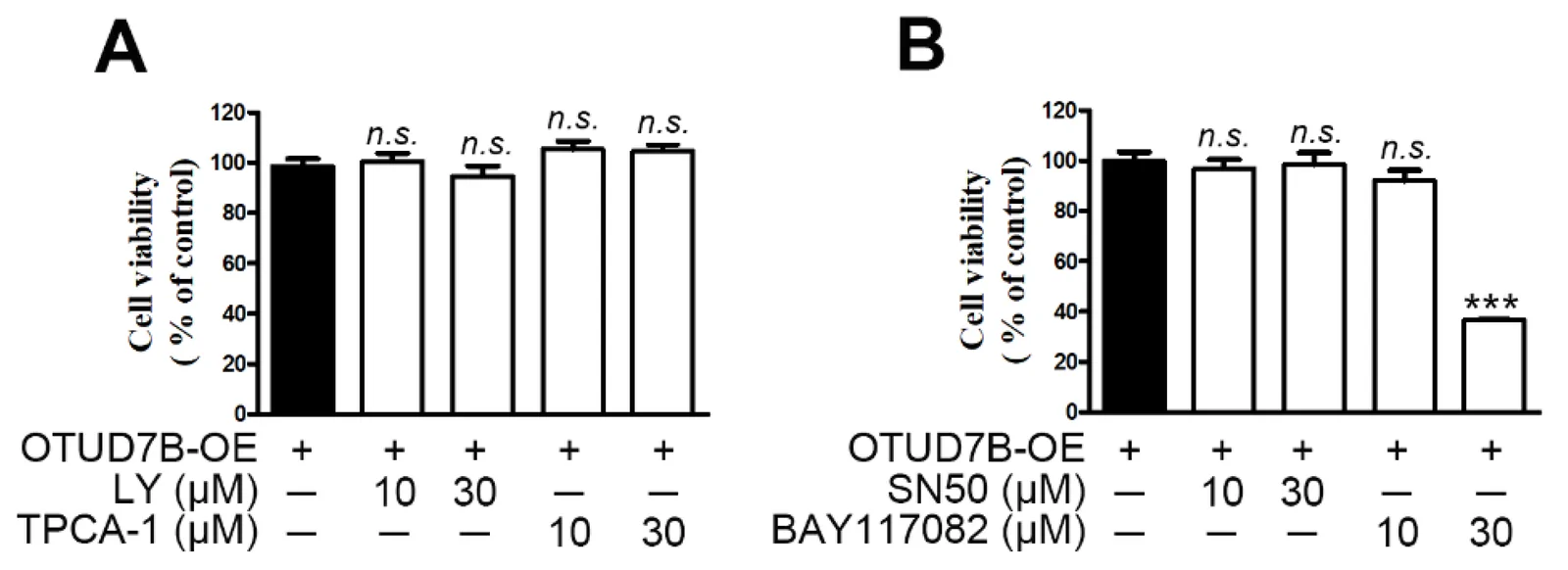
**Cell viability of OTUD7B-overexpressing (OTUD7B-OE) HCC1937 cells following NF-κB pathway inhibition.** (**A**,**B**) Cell viability of OTUD7B-OE HCC1937 cells was assessed using the CCK-8 assay after 36 h treatment with the IKKβ inhibitors LY2409881 (LY) and TPCA-1 (**A**), and the NF-κB inhibitors BAY11-7082 (BAY) and SN50 (**B**) at the indicated concentrations. n.s., not significant; ***p < 0.001 compared with the untreated group.

Because OTUD7B knockdown elevated the ubiquitinated protein levels of NEMO in Hs578T cells, we next perform Cycloheximide-chase assay to detect the protein degradation rate of NEMO. The data showed that OTUD7B knockdown, compared to non-silence control, accelerates the protein degradation of NEMO in Hs578T cells (Fig. 5A). In contrast, OTUD7B overexpression in HCC1937 cells enhanced the association between OTUD7B and NEMO (Fig. A8A,B). Since the previous report has demonstrated that Caspase-8-RIPK1-NEMO complex activates IKKα/β to trigger the activation of the NF-κB pathway [15], we further performed the pharmaceutical inhibition of IKKβ by its specific inhibitors LY2409881 and TPCA-1. The treatment with LY2409881 and TPCA-1 dose-dependently suppressed the OTUD7B-promoted migration ability of HCC1937 cells (Fig. 5B,C) without cytotoxicity (Fig. A9A). Accordingly, the pharmaceutical inhibition of NF-κB by its specific inhibitors BAY11-7082 and SN50 effectively mitigated the OTUD7B-promoted migration ability of HCC1937 cells in a dose-dependent manner (Fig. 5D,E). However, BAY11-7082 at 30 μM exhibited detectable cytotoxicity (Fig. A9B).

### 3.6 OTUD7B Knockdown Induces Necroptosis in TNBC Cells

**Figure A10:**
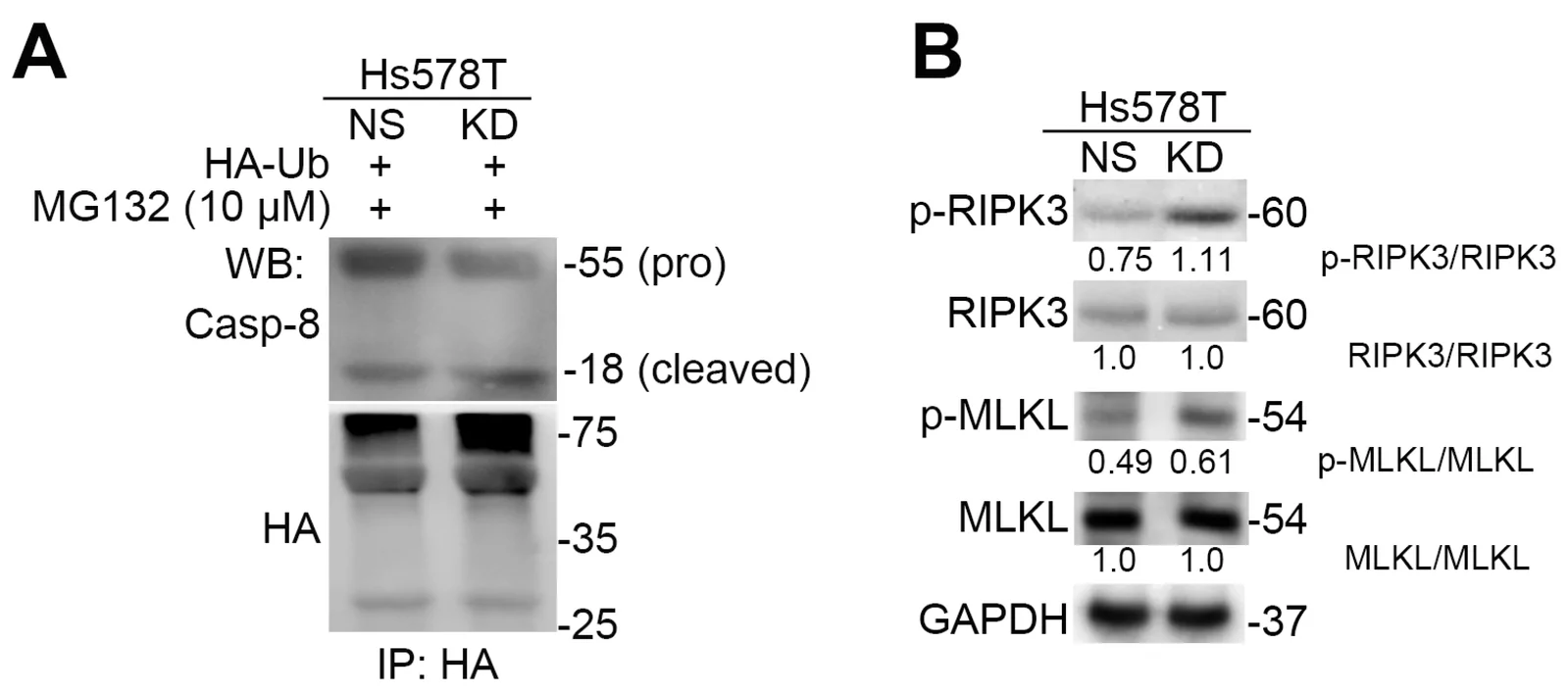
**Ubiquitination/activation of Caspase-8 and expression of necroptosis markers p-RIPK3 and p-MLKL following OTUD7B knockdown in Hs578T cells.** (**A**,**B**) Western blot analyses of immunoprecipitates obtained using an anti-HA antibody and subsequently probed with anti-Caspase-8 and anti-HA antibodies (**A**), and protein expression levels of phosphorylated RIPK3 (p-RIPK3)/total RIPK3 and phosphorylated MLKL (p-MLKL)/total MLKL in whole-cell lysates (**B**) derived from nonsilencing (NS) control and OTUD7B-knockdown (OTUD7B-KD) Hs578T cells. GAPDH also served as the loading control in (**B**).

**Figure A11:**
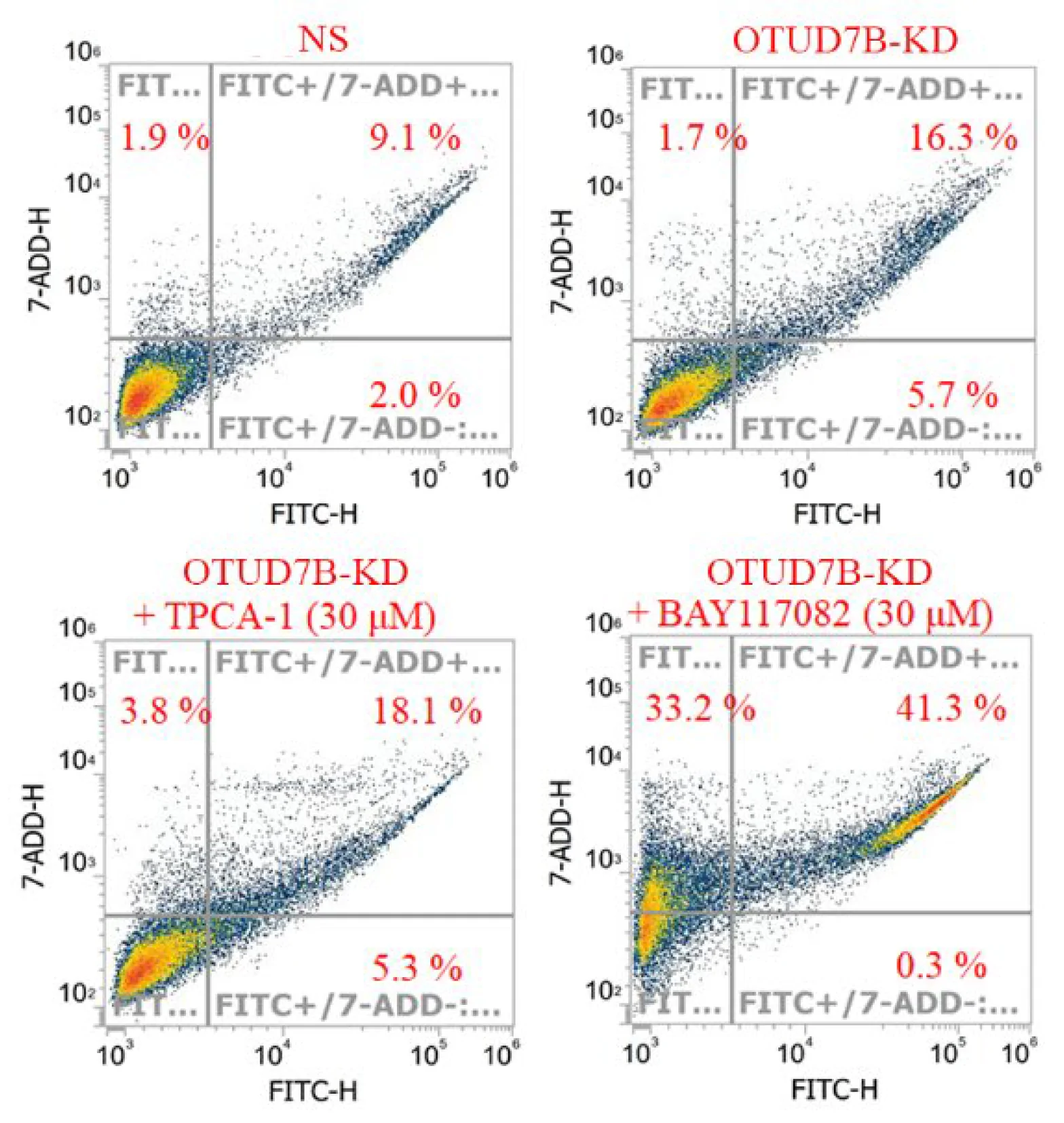
Cell necroptosis in OTUD7B-KD Hs578T cells in the presence of IKKβ and NF-κB inhibitors. Flow cytometric analysis using 7-AAD (*y*-axis) and Annexin V (*x*-axis) staining was performed in nonsilencing (NS) control and OTUD7B-knockdown (OTUD7B-KD) Hs578T cells, with or without treatment with the IKKβ inhibitor TPCA-1 or the NF-κB inhibitor BAY11-7082 (30 μM, 16 h).

The destruction of the Caspase-8-RIPK1-NEMO complex has been shown to induce necroptosis [16]. Here we found that OTUD7B knockdown in Hs578T cells dramatically enhances the pro (Annexin V+/PI−)- and late (Annexin V+/PI+)-apoptotic cells (Fig. 5F). In addition, OTUD7B knockdown in Hs578T cells increased ubiquitinated cleaved (active) caspase-8 (Fig. A10A) and elevated levels of p-RIPK3 and p-MLKL, two established markers of necroptosis activation [17,18], supporting the induction of the RIPK3–MLKL necroptotic axis upon OTUD7B depletion (Fig. A10B). The treatments with pan-caspase inhibitor Z-VAD or necroptosis inhibitor Nec-1 dose-dependently decreased the pro- and late-apoptotic populations in the OTUD7B-knockdown Hs578T cells (Fig. 5F). In contrast, the treatment with IKKβ inhibitor TPCA-1 and NF-κB inhibitor BAY11-7082 in OTUD7B-knockdown Hs578T cells further enhanced necroptotic cell death (Fig. A11). Re-expression of NEMO (Fig. 5G,H) and inhibition of necroptosis by Nec-1 (Fig. 5I,J) markedly restored the activity of NF-κB, as evidenced by increased phosphorylation levels of NF-κB signaling proteins, and rescued the migratory ability of OTUD7B-knockdown Hs578T cells.

## 4 Discussion

**Figure 6:**
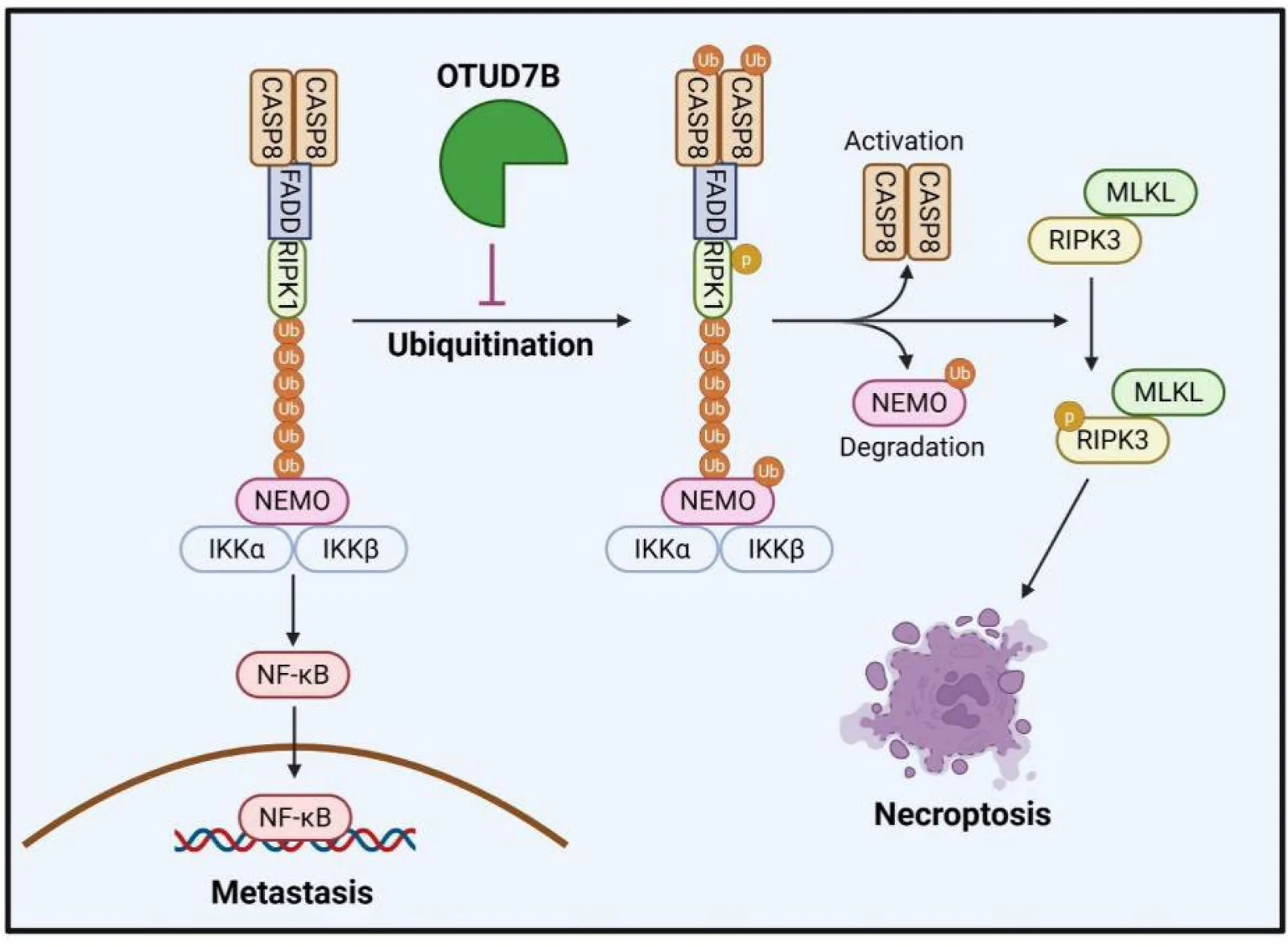
The proposed mechanism in which OTUD7B prevents TNBC cells from necroptosis by inhibiting Caspase-8/NEMO ubiquitination and thereby stabilizes Caspase-8-RIPK1-NEMO complex to activate NF-κB-regulated transcription of metastasis-related genes. The figure was generated by using BioRender software.

Distant metastasis is commonly found and refers to a poor outcome in TNBC patients. Here we find that the upregulation of deubiquitinase OTUD7B is dominant in primary tumors compared with normal mammary tissues and correlates with an increased risk for distant metastasis in TNBC patients. Our data further showed that OTUD7B probably prevents the ubiquitination of Caspase-8 via removing K48-linked ubiquitin chains and thereby stabilizes its complex with RIPK1 and NEMO (Fig. A6A), which ultimately activates the NF-κB pathway to force the metastatic progression of TNBC (Fig. 6). Conversely, targeting the deubiquitinating activity of OTUD7B may promote the ubiquitination and proteolytic activation of Caspase-8, which leads to the induction of necroptotic cell death of TNBC (Fig. 6). These findings suggest that OTUD7B could be a biomarker for predicting distant metastasis but also serve as a new therapeutic target for combating metastatic TNBCs.

The addition of ubiquitin to a target protein was found to be either mono-(monoubiquitination) or poly-(polyubiquitination) conjugates that are tethered to any of the seven lysine residues (Lys6, 11, 27, 29, 33, 48, and 63) or through the N-terminal methionine residue in the first ubiquitin molecule. Whereas monoubiquitination usually prevents target protein from proteasomal degradation, polyubiquitination formed with distinct linkages of these ubiquitin residues is commonly recognized by cellular proteasome machinery [19,20]. OTUD7B has been shown to have similar binding activity towards Lys11, 48, and 63 and a higher catalytic activity on Lys11 of ubiquitin [13]. Intriguingly, Lys48-linked polyubiquitination typically results in proteasomal degradation of the target protein; whereas Lys63-linked polyubiquitination is the best-studied non-proteolytic ubiquitination, which regulates the enzymatic activity (e.g., kinase activity) [21] or subcellular localization [22] of target proteins. It has been known that Caspase-8 and RIPK1 ubiquitination is formed by Lys48 and Lys63-linked polyubiquitination, respectively [23,24,25,26]. Our data revealed that OTUD7B knockdown enhances Caspase-8 ubiquitination but reduces RIPK1 ubiquitination (Fig. A3A). This discrepancy is probably attributed to the difference in the branch of polyubiquitination and the compensation of other similar deubiquitinases, e.g., A20 [27,28], after OTUD7B knockdown.

Caspase-8 has been shown to play two distinct roles in response to TRAIL receptor engagement as a scaffold for assembly of a Caspase-8-FADD-RIPK1 complex, leading to NF-κB-dependent inflammation, or as a protease that promotes apoptosis [29]. The Lys48-linked polyubiquitination of Caspase-8 is required for its dimerization and the initiation of extrinsic caspase-dependent apoptosis [28,29,30]. Here we find that OTUD7B knockdown enhances the ubiquitination of Caspase-8 and promotes cell death of Hs578T cells through necroptotic machinery. Conversely, OTUD7B overexpression reduces cell necroptosis, even though the protein levels of ubiquitinated Caspase-8 need to be further explored. Therefore, our results might imply that OTUD7B prevents the ubiquitination of Caspase-8 to stabilize Caspase-8-FADD-RIPK1 complex, which may ultimately trigger the NF-κB-mediated metastatic progression of TNBCs.

The Lys63-linked polyubiquitination of RIPK1 was shown to be critical for the recruitment of NEMO and the subsequent activation of IKKα/β complex, which further activates NF-κB via phosphorylating I-κB and promoting its proteasomal degradation [31]. Here, we found that OTUD7B knockdown elevates the ubiquitination of NEMO and accelerates its protein degradation in Hs578T cells, which might indicate NEMO as an OTUD7B target. The pharmaceutical inhibition of IKKβ and NF-κB predominantly suppressed the metastatic potentials of TNBC cells, indicating that NF-κB activation through the regulation by Caspase-8-RIPK1-NEMO axis is crucial for the OTUD7B-promoted TNBC metastasis. One limitation of the present study is that although our findings support a role for OTUD7B in regulating the Caspase-8–RIPK1–NEMO signaling axis, the precise ubiquitin linkage types and modification sites involved were not fully characterized. Given that OTUD7B exhibits distinct catalytic preferences toward different polyubiquitin chains, additional studies utilizing ubiquitin linkage-specific assays and mass spectrometry-based analyses will be required to define the exact ubiquitination events regulated by OTUD7B in TNBC cells. In addition, although our data suggest that OTUD7B stabilizes the Caspase-8-containing signaling complex and promotes NF-κB activation, the direct biochemical interaction between OTUD7B and each signaling component requires further validation. Future studies using purified proteins, catalytic mutants, or reconstitution systems may help clarify whether these proteins are bona fide direct substrates of OTUD7B.

## 5 Conclusions

Deubiquitinases (DUBs) have emerged as promising therapeutic targets, with several DUB inhibitors demonstrating potential for cancer treatment. Nevertheless, further studies are required to enhance their selectivity and efficacy and to advance our understanding of DUB regulation for the development of next-generation inhibitors [32]. Collectively, this study is the first to document that deubiquitinating enzyme OTUD7B is capable of fostering TNBC metastasis via preventing Caspase-8 ubiquitination and thereby inducing the NF-κB-regulated transcription. Our findings also provide a new therapeutic strategy to combat TNBC by targeting the deubiquitinating activity of OTUD7B.

## Data Availability

The data presented in this study are available on request from the corresponding authors.

## Abbreviations

TNBC	Triple-negative breast cancer
ER	Estrogen receptor
PR	Progesterone receptor
HER2	Human epidermal growth factor receptor 2
OTUD7B	Human ovarian tumor deubiquitinating enzyme 7B
TCGA	The Cancer Genome Atlas
MOI	Multiplicity of infection
EGFR	Epidermal growth factor receptor
RIPK1	Receptor-interacting serine/threonine-protein kinase 1
IKK	IKB kinase

## Appendix A

**Table A1:** Cox univariate and multivariate analyses under the condition of overall survival probability in association with OTUD7B mRNA expression levels and pathological stage derived TCGA cohort with TNBC (*n* = 123). HR: hazard ratio; CI: confidence interval; NA, not applicable.

Variables	Crude HR (95%CI)	*p*	Adjusted HR (95%CI)	*p*
Age				
≤60	1	NA	1	NA
>60	1.24 (0.47–3.26)	0.668	1.36 (0.48–3.91)	0.546
Pathologic T stage				
I–II	1	NA	1	NA
III–IV	3.15 (1.13–8.81)	0.029	1.06 (0.32–3.56)	0.925
Nodal status				
Negative	1	NA	1	NA
Positive	3.79 (1.44–9.97)	0.007	6.65 (1.74–25.4)	0.006
Radiotherapy				
No	1	NA	1	NA
Yes	0.47 (0.18–1.12)	0.113	0.15 (0.04–0.53)	0.003
OTUD7B expression				
low	1	NA	1	NA
high	3.36 (1.11–10.2)	0.032	3.17 (0.97–10.4)	0.057
